# Systemic dual-gene therapy reverses biochemical intoxication in the central metabolic compartment of *Bckdha−/−* mice

**DOI:** 10.1016/j.ymthe.2026.05.008

**Published:** 2026-05-13

**Authors:** Jiaming Wang, Coleman T. Turgeon, Perry R. Loken, Heather Gray-Edwards, Guangping Gao, Silvia Tortorelli, Dan Wang, Kevin A. Strauss

**Affiliations:** 1 Department of Genetic and Cellular Medicine, University of Massachusetts Chan Medical School, Worcester, MA, USA; 2 Biochemical Genetics Laboratory, Department of Laboratory Medicine and Pathology, Mayo Clinic, Rochester, MN, USA; 3 Tufts University Cummings School of Veterinary Medicine, North Grafton, MA, USA; 4 Department of Microbiology and Physiological Systems, University of Massachusetts Chan Medical School, Worcester, MA, USA; 5 RNA Therapeutics Institute, University of Massachusetts Chan Medical School, Worcester, MA, USA; 6 Clinic for Special Children, Gordonville, PA, USA; 7 Plowshare Therapies, Lancaster, PA, USA

**Keywords:** 2-ketoisocaproic acid, AAV, gene therapy, metabolomics, MSUD

## Abstract

Branched-chain 2-ketoacid dehydrogenase (BCKDH) deficiency (maple syrup urine disease; MSUD) causes lethal encephalopathy by disrupting cerebral metabolism, a process imperfectly reflected by circulating biomarkers. Diet and liver transplantation stabilize peripheral metabolites but fail to restore brain neurochemistry, demarcating the central nervous system as the decisive therapeutic compartment. To define the pathogenesis of intoxication and its therapeutic response, we performed paired serum-brain metabolomics in *Bckdha−/−* mice treated with a systemic AAV9 dual-gene vector encoding human *BCKDHA* and *BCKDHB* (A-BiP-B). Untreated neonates exhibited a 9-fold elevation of brain 2-ketoisocaproate accompanied by cerebral depletion of glutamate and glutamine, as well as shifts in tricarboxylic acid cycle and ketone body metabolism. These disturbances originated from reversal of branched-chain aminotransferase 2 flux and destabilization of glutamate-2-ketoglutarate mass balance, producing divergent metabolic endophenotypes in blood versus brain. A single intravenous injection of A-BiP-B rescued mice from fatal encephalopathy, partially restored cerebral *BCKDHA* mRNA expression, and brought core brain neurochemical endpoints within wild-type range despite persistent elevation of serum 2-ketoacids. These findings expose limitations of current MSUD management and establish systemic dual-gene therapy as a means of restoring neurochemical homeostasis while enabling survival on unrestricted protein intake.

## Graphical abstract

**Figure:**
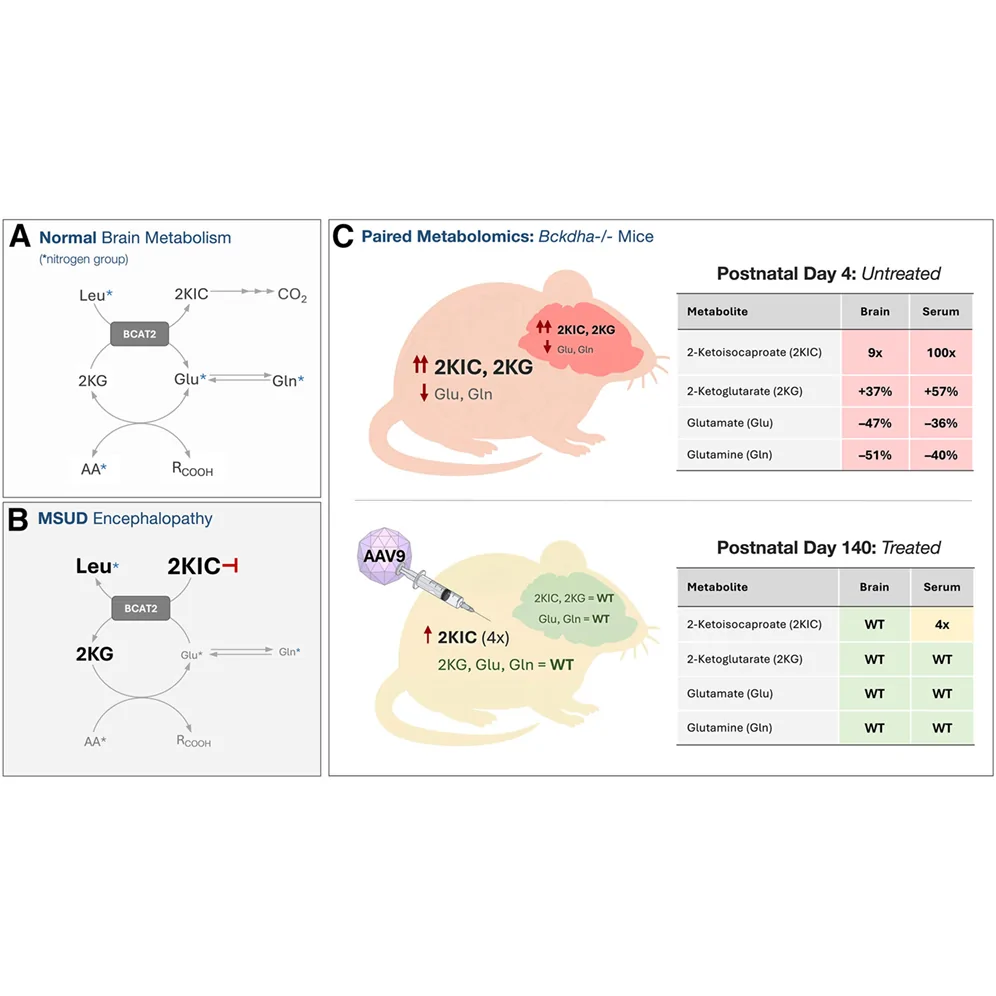

## Teaser

In *Bckdha−/−* mice, systemic AAV9 dual-gene therapy normalizes brain neurochemistry despite residual peripheral metabolic abnormalities. Paired serum-brain metabolomics reveals that 2-ketoisocaproate disrupts cerebral neurotransmitter and energy homeostasis through mass-action reversal of transamination equilibria. Partial restoration of central nervous system enzyme activity stabilizes this network, establishing the brain as the decisive therapeutic compartment in maple syrup urine disease.

## Introduction

Branched-chain 2-ketoacid dehydrogenase (BCKDH) deficiency, commonly known as maple syrup urine disease (MSUD), is marked by neurotoxic accumulation of branched-chain amino acids (BCAAs) and their 2-ketoacids (BCKAs), especially leucine and 2-ketoisocaproic acid (2KIC).^1^^,^^2^ Without prompt intervention, neonates with the severe (classic) form lapse into coma within days and die from metabolic encephalopathy.^3^ Despite vigilant treatment, survivors face recurrent crises, progressive brain injury, and mental illness.^4^^,^^5^

MSUD encephalopathy has both chronic and acute dimensions.^5^ Hyperleucinemia blocks cerebral uptake of essential amino acids, disrupting protein and neurotransmitter synthesis and potentially stunting brain development.^6^^,^^7^^,^^8^ By contrast, high cerebral 2KIC appears to drive the more destructive neurochemistry of acute intoxication.^9^^,^^10^ Patients with branched-chain amino transaminase 2 (BCAT2) deficiency vividly illustrate this distinction: despite BCAA elevations in the MSUD range, they present with milder symptoms of later onset and, in the absence of elevated BCKAs, never experience intoxication.^11^ These observations suggest that peripheral biomarkers do not fully reflect the biochemical processes that govern neurological injury.^5^^,^^12^

Consistent with this view, neurochemical, cognitive, and affective disturbances persist in MSUD patients under good peripheral metabolic “control,” signaling the existence of distinct peripheral and central biochemical compartments. Muelly et al. identified cerebral deficits of glutamate, creatine, and N-acetylaspartate in stable MSUD patients with enduring neuropsychiatric symptoms after liver transplantation.^5^ Data from conditional *Dbt* knockout mice further explain these findings: despite stable peripheral BCAAs, brain-specific BCKDH deficiency is associated with 50%–90% elevations of cerebral BCAAs accompanied by abnormal cortical activity, epileptic spikes, and anxious behavior.^12^ Together, these data indicate that complete neurological protection from MSUD requires restoration of BCKDH activity in peripheral tissues and the central nervous system (CNS).

Recombinant adeno-associated viruses (AAVs) are well suited to this task.^13^^,^^14^ Systemic AAV delivery can restore human *BCKDHA* and *BCKDHB* expression across tissues, including brain.^15^^,^^16^^,^^17^ However, efficacy in prior studies has been judged largely by plasma BCAA levels—peripheral surrogates that can conceal ongoing CNS derangements.^5^^,^^12^ Here, we show that systemic administration of an AAV9 vector encoding codon-optimized human *BCKDHA* and *BCKDHB* (A-BiP-B; Figure S1) partially restores cerebral *BCKDHA* mRNA expression, returns CNS neurotransmitter and energy-related metabolites to the wild-type range, and rescues *Bckdha−/−* mice from fatal encephalopathy despite residual elevation of serum 2-ketoacids. We conclude that partial restoration of CNS BCKDH activity is sufficient to stabilize the cerebral glutamate-energy network in MSUD, and that systemic dual-gene therapy can restore neurochemical homeostasis while enabling survival on unrestricted protein intake.

## Results

### A neonatal knockout model of MSUD

To model MSUD, we used homozygous *Bckdha*−/− mice recovered from a cryopreserved line carrying an intron-exon disruption.^15^^,^^17^ All procedures were approved by the Institutional Animal Care and Use Committee of UMass Chan Medical School and conformed to relevant regulations (PROTO202000184). Consistent with the natural history of classic MSUD,^3^^,^^18^ untreated neonatal mice exhibited impaired growth, metabolic intoxication, CNS vacuolation, and death by postnatal day 12 (P12).

### Experimental cohorts and metabolomic profiling

We studied four cohorts (*n* = 3 female mice per group): postnatal day 4 (P4) wild-type (WT) vs. P4 *Bckdha−/−* (MSUD), and P140 WT vs. P140 *Bckdha−/−* treated with A-BiP-B. Age-matched controls were used to accommodate maturational changes of mouse brain chemistry,^19^ and groups were co-housed to minimize cage effects. No animals were excluded. Brains were collected after PBS perfusion and then snap-frozen and stored at −80°C. Serum and brain tissue were harvested simultaneously. Metabolites were quantified by LC–MS/MS (amino acids) and GC–MS (organic acids) using stable-isotope standards. Data are reported in conventional units, with model fits using linear or one-phase decay according to data shape. This study was designed as a paired serum-brain targeted metabolomics analysis in a neonatal-lethal model, prioritizing depth of biochemical phenotyping within each compartment. Primary endpoints are biochemical and survival based; formal neurobehavioral assessments were not performed.

### Neurochemical compartmentalization in MSUD intoxication

**Figure 1:**
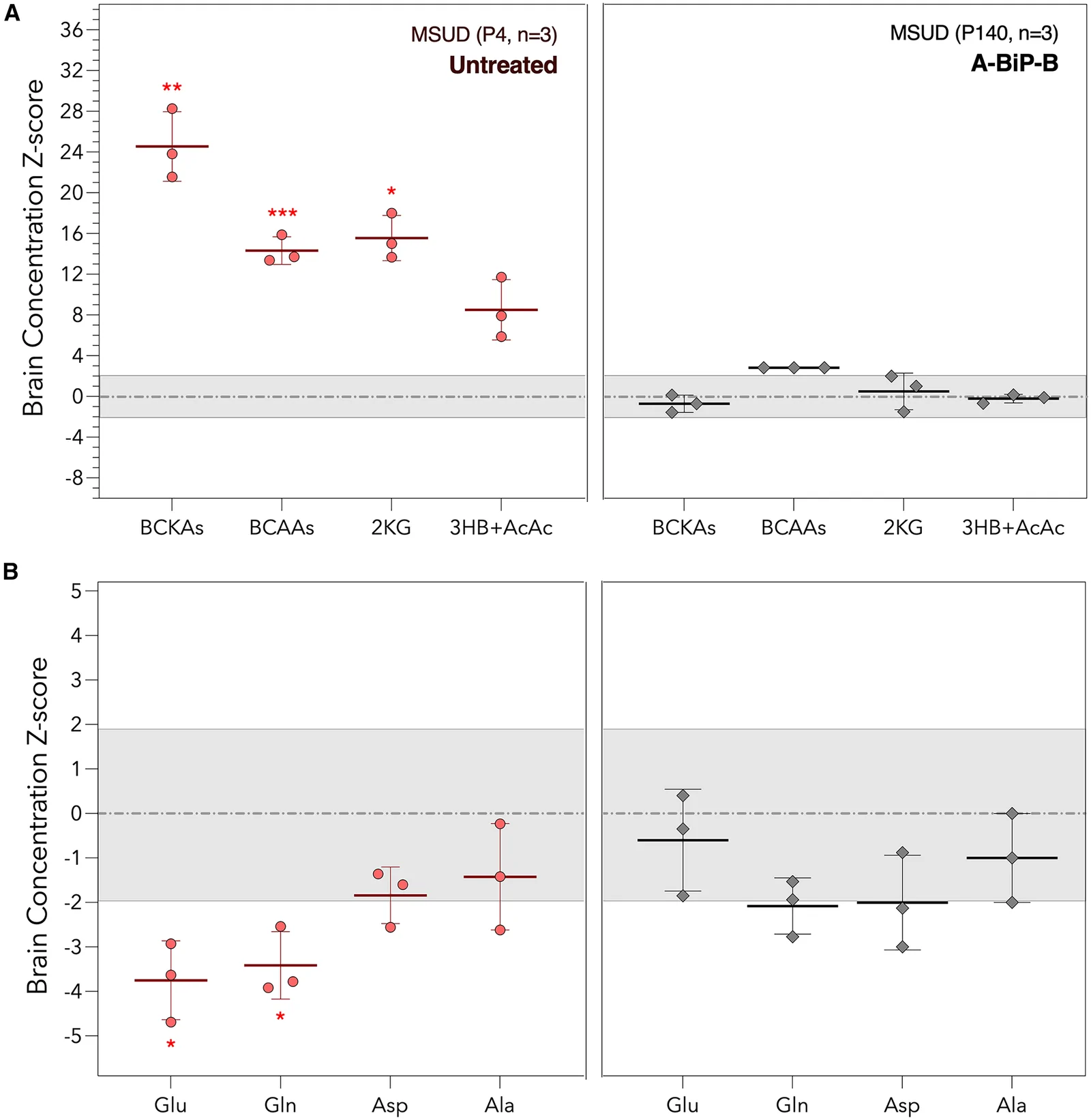
Brain biomarkers in untreated and vector-treated *Bckdha−/−* mice Metabolites were measured in brain tissue of P4 untreated (left panels, red circles) and P140 vector-treated (A-BiP-B; right panels, gray diamonds) *Bckdha−/−* mice. (A) BCAAs (Leu + Ile + Val), BCKAs (2KIC + 3M2KV + 2KIV), ketone bodies (3HB + AcAc), and 2KG were elevated in brain tissue from intoxicated P4 MSUD mice. (B) These changes were accompanied by 47%–51% lower brain Glu and Gln (q < 0.05). Aspartate showed a biologically consistent 32% reduction that did not reach significance after global FDR correction. Right panels show brain metabolite values in P140 A-BiP-B-treated *Bckdha−/−* mice relative to age-matched wild-type controls. Data are plotted as Z scores in relation to age-matched wild-type control mice. Multiple, unpaired, two-tailed t tests with Welch correction, FDR 5%: q∗<0.05; q∗∗<0.01; q∗∗∗<0.001. 2KG, 2-ketoglutarate; 3HB, 3-hydroxybutyrate; AcAc, acetoacetate; Ala, alanine; Asp, aspartate; BCAAs, branched-chain amino acids; BCKAs, branched-chain ketoacids; Gln, glutamine; Glu, glutamate.

**Figure 2:**
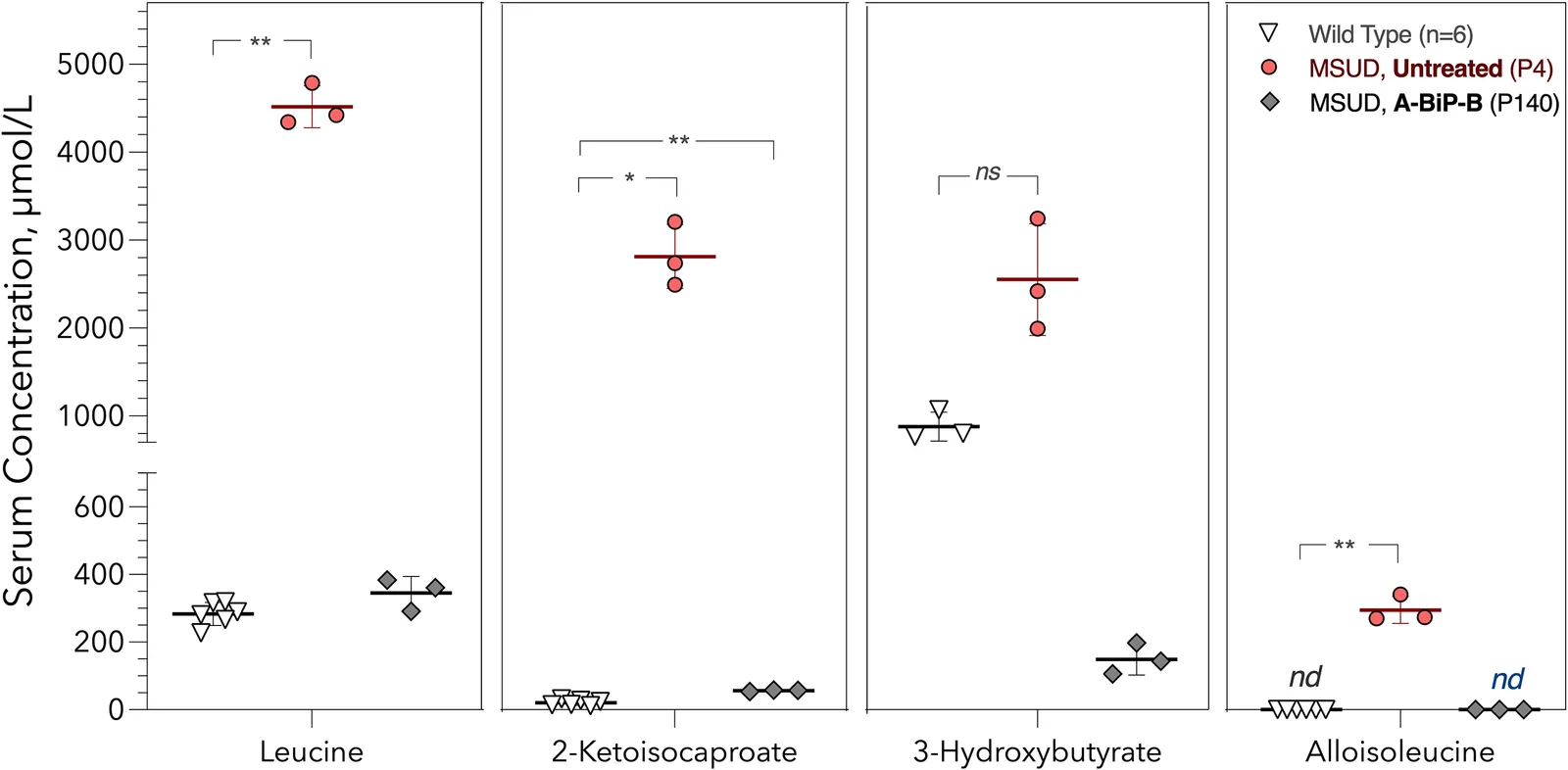
Serum biomarkers in untreated and vector-treated *Bckdha−/−* mice MSUD biomarkers were markedly elevated in sera of P4 MSUD mice (red circles) as compared with their wild-type counterparts (white triangles) and improved following a single intravenous dose of AAV9 h*BCKDHA*-h*BCKDHB* replacement therapy (A-BiP-B; gray diamonds), with residual BCKA elevations at P140 (Table S2).We examined *n* = 3 per group at each time point; exact values are in Tables S1 and S2. Multiple, unpaired, two-tailed t tests with Welch correction, FDR 5%: ∗q < 0.05; ∗∗<0.01. MSUD, maple syrup urine disease; nd, not detected.

**Figure 3:**
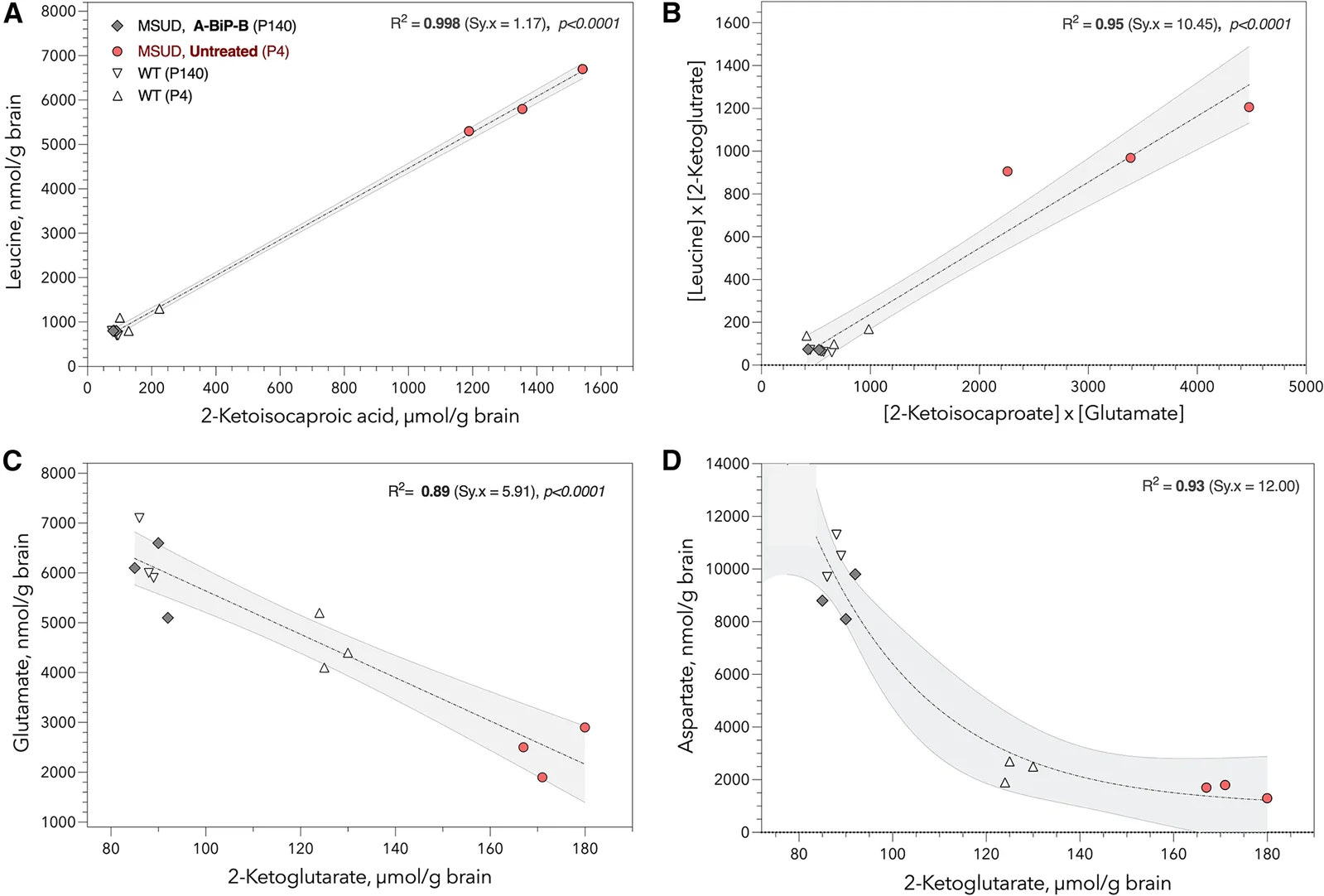
Neurochemical relationships across wild-type and *Bckdha−/−* mouse brains Correlation plots (A)–(D) depict all four experimental cohorts composed of three animals per cohort: P4 WT (upward white triangles), P140 WT (downward white triangles), P4 MSUD untreated (red circles), and P140 MSUD A-BiP-B-treated (gray diamonds) mice. Correlations are shown across all four cohorts combined (*n* = 12); within-cohort analyses and Spearman ρ are reported in Table S6. (A) The relationship between 2KIC and leucine in brain tissue was linear (R^2^ = 0.998, Spearman ρ = 0.74, *p* < 0.0001), and (B) BCAT mass action was conserved across groups (R^2^ = 0.95, *p* < 0.0001), represented here as the product of products (leucine × 2KG) plotted against the product of reactants (2KIC × glutamate). (C) We found an inverse correlation between 2KG and glutamate (R^2^ = 0.89, Spearman ρ = −0.91, *p* < 0.0001) reflected by a 3-fold change of their concentration ratio in brain and serum of P4 MSUD mice. (D) This mass shift altered other transamination equilibria, such as the glutamate-oxaloacetate transaminase (GOT2) reaction that controls tissue concentrations of aspartate (R^2^ = 0.80, Spearman ρ = −0.84, *p* = 0.0006). These secondary effects adhered to one-phase decay models and are more fully depicted in Figure 4. Gray shading indicates the 95% confidence interval.

Intoxicated *Bckdha*−/− neonates had markedly elevated BCKAs and BCAAs in brain and serum (Tables 1 and 2; Tables S1 and S2; Figures 1 and 2). 2KIC and leucine comprised 70% and 53% of cerebral pools, respectively, and their concentrations were tightly linked (R^2^ > 0.99, *p* < 0.0001; Figure 3A). Mass-action analysis verified the reversal of BCAT2 transamination in tissues, conforming to the law of Guldberg and Waage (R^2^ = 0.95; Figures 3B and 3C),^20^ where

(Equation 1) BCAT1/2:Leucine+2KG⇌Glutamate+2KIC

(Equation 2) $$K_{\text{BCAT}1/2}=\frac{\left[\text{Glutamate}\right]\times \left[2\text{KIC}\right]}{\left[\text{Leucine}\right]\times \left[2\text{KG}\right]}$$

**Table 1:** Brain metabolites in wild-type and *Bckdha−/−* mice, postnatal day 4: Untreated

Metabolite	Wild type (*n* = 3)				MSUD, untreated (*n* = 3)				Statistics			
	Units	Mean	SD	SEM	Mean	SD	SEM	Δ%	*p* value	q value	Discovery^a^	Hedges’g
**Core BCKDH-BCAT module**												

Leucine	nmol/g	**1,067**	252	145	**5,933**	709	410	456	0.0034	**0.0131**	**yes**	7.31
Isoleucine	nmol/g	**567**	153	88.2	**2,033**	115	66.7	259	0.0003	**0.0059**	**yes**	8.67
Valine	nmol/g	**900**	200	115	**3,200**	100	57.7	256	0.0004	**0.0059**	**yes**	11.64
2-Ketoisocaproate	μmol/g	**151**	64.6	37.3	**1,362**	177	102	802	0.0033	**0.0131**	**yes**	7.27
2-Keto-3-methyl-N-valerate	μmol/g	**68.7**	3.1	1.8	**308**	52.0	30.0	348	0.0152	**0.0409**	**yes**	5.19
2-Ketoisovalerate	μmol/g	**19.0**	2.6	1.5	**287**	17.8	10.3	1,411	0.0012	**0.0107**	**yes**	16.87
2-Ketoglutarate	μmol/g	**126**	3.2	1.9	**173**	6.7	3.8	37	0.0020	**0.0131**	**yes**	7.09
Glutamate	nmol/g	**4,567**	569	328	**2,433**	503	291	−47	0.0086	**0.0289**	**yes**	−3.18
Glutamine	nmol/g	**4,833**	723	418	**2,367**	551	318	−51	0.0110	**0.0329**	**yes**	−3.07

**Transamination and energy substrates**												

Aspartate	nmol/g	**2,367**	416	240	**1,600**	265	153	−32	0.0651	0.1599	no	−1.76
Alanine	nmol/g	**3,433**	586	338	**2,600**	700	404	−24	0.1912	0.2868	no	−1.03
Serine	nmol/g	**2,200**	400	231	**1,667**	153	88.2	−24	0.1348	0.2426	no	−1.41
GABA	nmol/g	**2,533**	723	418	**2,867**	208	120	13	0.5132	0.6299	no	0.50
Citrate	μmol/g	**400**	133	76.7	**608**	117	67.5	52	0.1126	0.2172	no	1.33
Succinate	μmol/g	**186**	74.1	42.8	**253**	116	67.2	36	0.4599	0.5913	no	0.54
Fumarate	μmol/g	**382**	99.0	57.2	**368**	86.1	49.7	−4	0.8626	0.9316	no	−0.12
Malate	μmol/g	**1,345**	194	112	**1,213**	386	223	−10	0.6340	0.7443	no	−0.35

**Redox pairs**												

Lactate	μmol/g	**13,884**	1,106	638	**11,787**	1,221	705	−15	0.0927	0.2007	no	−1.44
Pyruvate	μmol/g	**542**	308	178	**563**	145	83.9	4	0.9220	0.9575	no	0.07
3-Hydroxybutyrate	μmol/g	**51.7**	52.9	30.6	**289**	203	117	460	0.1730	0.2747	no	1.28
Acetoacetate	μmol/g	**10.7**	0.58	0.33	**23.0**	7.2	4.2	116	0.0966	0.2007	no	1.93

**SLC7A5 transporter competitors**												

Histidine	nmol/g	**533**	115	66.7	**500**	0.00	0.00	−6	0.6667	0.7500	no	−0.33
Methionine	nmol/g	**367**	57.7	33.3	**400**	0.00	0.00	9	0.4226	0.5706	no	0.65
Phenylalanine	nmol/g	**400**	100	57.7	**400**	0.00	0.00	0	1.0000	1.0000	no	0.00
Threonine	nmol/g	**1,500**	200	115	**1,267**	115	66.7	−16	0.1727	0.2747	no	−1.14
Tyrosine	nmol/g	**500**	100	57.7	**433**	57.7	33.3	−13	0.3868	0.5497	no	−0.65

**Other relevant metabolites**												

Alloisoleucine	nmol/g	**N/D**			**367**	57.7	33.3		N/A			
Taurine	nmol/g	**22,600**	781	451	**28,400**	265	153	26	0.0030	**0.0131**	**yes**	7.96

BCAT, branched-chain amino acid transaminase; BCKDH, branched-chain 2-ketoacid dehydrogenase complex; GABA, 4-aminobutyric acid; MSUD, maple syrup urine disease; N/A, not applicable; N/D, not detected; SD, standard deviation; SEM, standard error of the mean; Δ%, percent difference from wild-type.

a Multiple, unpaired, two-tailed t tests with Welch correction (assuming individual variances), false discovery rate (FDR) set at 5%. The q value represents a *p* value corrected for multiple comparisons.

**Table 2:** Brain metabolites in wild-type and *Bckdha−/−* mice, postnatal day 140: Treated with AAV9-A-BiP-B

Metabolite	Wild-type (*n* = 3)				MSUD, treated (*n* = 3)				Statistics			
	Units	Mean	SD	SEM	Mean	SD	SEM	Δ%	*p* value	q value	Discovery^a^	Hedges’ g
**Core BCKDH-BCAT module**												

Leucine	nmol/g	**733**	57.7	33.3	**800**	0.00	0.00	9	0.1835	0.7078	no	1.31
Isoleucine	nmol/g	**267**	57.7	33.3	**400**	0.00	0.00	50	0.0572	0.4065	no	2.61
Valine	nmol/g	**367**	57.7	33.3	**600**	0.00	0.00	64	0.0198	0.4065	no	4.57
2-Ketoisocaproate	μmol/g	**87.3**	10.0	5.8	**84.7**	5.0	2.9	−3	0.7084	0.8492	no	−0.27
2-Keto-3-methyl-N-valerate	μmol/g	**65.7**	5.5	3.2	**63.7**	1.2	0.67	−3	0.5964	0.8115	no	−0.40
2-Ketoisovalerate	μmol/g	**4.0**	2.0	1.2	**3.7**	1.2	0.67	−8	0.8178	0.8492	no	−0.16
2-Ketoglutarate	μmol/g	**87.7**	1.5	0.88	**89.0**	3.6	2.1	2	0.6011	0.8115	no	0.39
Glutamate	nmol/g	**6,333**	666	384	**5,933**	764	441	−6	0.5323	0.8115	no	−0.45
Glutamine	nmol/g	**4,400**	721	416	**2,900**	458	265	−34	0.0476	0.4065	no	−1.99

**Transamination and energy substrates**												

Aspartate	nmol/g	**10,500**	800	462	**8,900**	854	493	−15	0.0773	0.4174	no	−1.55
Alanine	nmol/g	**1,900**	100	57.7	**1,800**	100	57.7	−5	0.2879	0.8115	no	−0.80
Serine	nmol/g	**1,667**	231	133	**1,567**	57.7	33.3	−6	0.5351	0.8115	no	−0.48
4-Aminobutyric acid (GABA)	nmol/g	**9,800**	1,217	702	**8,533**	850	491	−13	0.2215	0.7477	no	−0.97
Citrate	μmol/g	**695**	27.6	15.9	**632**	87.6	50.6	−9	0.3395	0.8115	no	−0.78
Succinate	μmol/g	**128**	25.0	14.4	**139**	16.1	9.3	9	0.5508	0.8115	no	0.43
Fumarate	μmol/g	**294**	38.4	22.2	**285**	9.5	5.5	−3	0.7277	0.8492	no	−0.26
Malate	μmol/g	**966**	19.9	11.5	**1,069**	51.8	29.9	11	0.0602	0.4065	no	2.10

**Redox pairs**												

Lactate	μmol/g	**14,345**	1,076	621	**15,413**	2,073	1,197	7	0.4858	0.8115	no	0.52
Pyruvate	μmol/g	**141**	83.2	48.1	**118**	82.9	47.8	−16	0.7550	0.8492	no	−0.22
3-Hydroxybutyrate	μmol/g	**36.0**	59.8	34.5	**25.7**	23.5	13.5	−29	0.8011	0.8492	no	−0.18
Acetoacetate	μmol/g	**12.3**	4.6	2.7	**10.7**	0.58	0.33	−14	0.5967	0.8115	no	−0.41

**SLC7A5 transporter competitors**												

Histidine	nmol/g	**433**	57.7	33.3	**400**	100	57.7	−8	0.6495	0.8350	no	−0.33
Methionine	nmol/g	**267**	57.7	33.3	**300**	0.00	0.00	13	0.4226	0.8115	no	0.65
Phenylalanine	nmol/g	**433**	57.7	33.3	**467**	57.7	33.3	8	0.5185	0.8115	no	0.46
Threonine	nmol/g	**733**	57.7	33.3	**700**	0.00	0.00	−5	0.4226	0.8115	no	−0.65
Tyrosine	nmol/g	**300**	100	57.7	**300**	0.00	0.00	0	1.0000	1.0000	no	0.00

**Other relevant metabolites**												

Alloisoleucine	nmol/g	**N/D**			**N/D**				N/A			
Taurine	nmol/g	**9,500**	1,480	854	**11,733**	451	260	24	0.1101	0.4957	no	1.63

BCAT, branched-chain amino acid transaminase; BCKDH, branched-chain 2-ketoacid dehydrogenase complex; GABA, 4-aminobutyric acid; MSUD, maple syrup urine disease; N/A, not applicable; N/D, not detected; SD, standard deviation; SEM, standard error of the mean; Δ%, percent difference from wild-type.

a Multiple, unpaired, two-tailed t tests with Welch correction (assuming individual variances), false discovery rate (FDR) set at 5%. The q value represents a *p* value corrected for multiple comparisons.

These correlations span the full experimental range across cohorts and may partly reflect between-group separation; within-cohort analyses are provided in Table S6. Accordingly, 9-fold elevation of cerebral 2KIC (Hedges’ g = +7.3) drove a 47% reduction of glutamate (g = −3.2), 37% increase of 2-ketoglutarate (2KG) (g = +7.1), and a 3-fold shift in their molar ratio, forcing glutamate oxidation via the tricarboxylic acid (TCA) cycle (Table S3). Elevations of 2KG correlated inversely with aspartate and alanine (Figure 3D), reflecting downstream equilibrium shifts for glutamate-oxaloacetate transaminase 2 (GOT2) and glutamate-pyruvate transaminase 2 (GPT2), respectively:

(Equation 3) GOT2:Glutamate+Oxaloacetate⇌2KG+Aspartate

(Equation 4) GPT2:Glutamate+Pyruvate⇌2KG+Alanine

Brain aspartate and alanine showed biologically consistent reductions (−32% and −24%, respectively; Hedges’ g = −1.8 and −1.0) that did not reach statistical significance after global false discovery rate (FDR) correction (q = 0.16 and 0.29), consistent with their position as secondary equilibrium effects downstream of the primary 2KG/glutamate shift.

**Figure 4:**
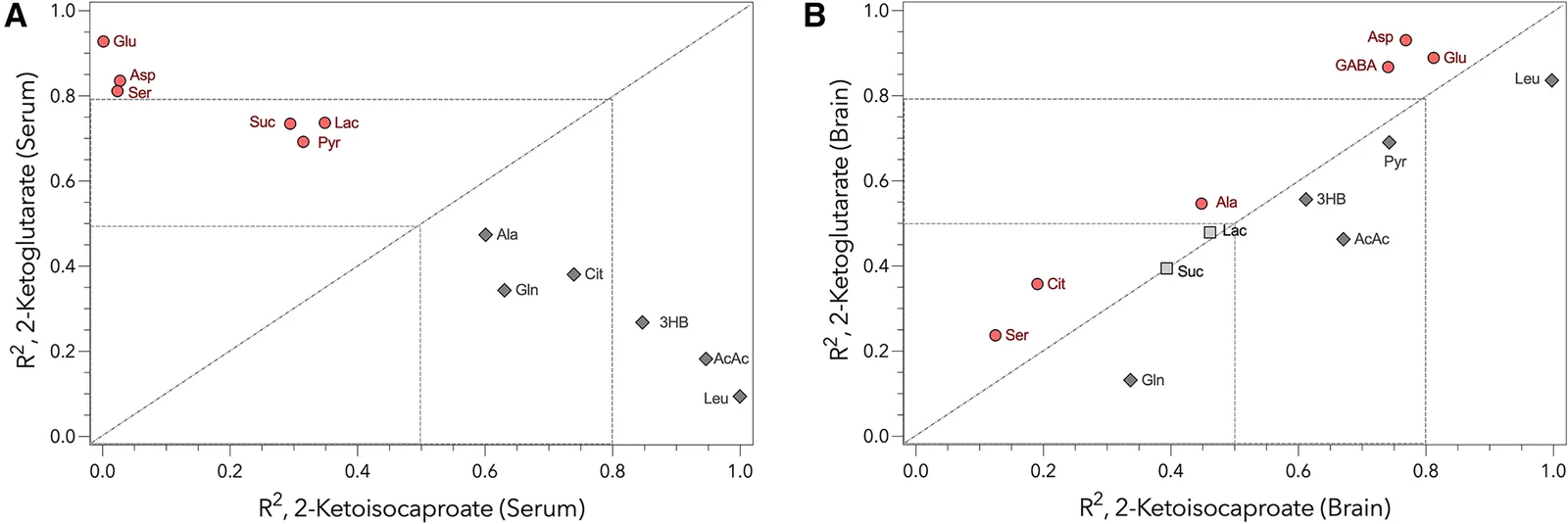
Correlation of 2-ketoisocaproate and 2-ketoglutarate with metabolites in separate blood and brain compartments Using one-phase decay, we modeled the correlation of various metabolites to 2KIC and 2KG in brain and serum. 2KIC and 2KG correlation coefficients (R^2^) were plotted against one another, where the diagonal represents unity, and gray dashed vertical-horizontal heuristic lines at R^2^ ≥ 0.5 and R^2^ ≥ 0.8 signify moderate and strong correlations, respectively. Symbols indicate if a metabolite is associated more strongly with 2KG (red circles), 2KIC (gray diamonds), or equally (white squares). The variable pattern of associations between blood and brain suggests a degree of metabolic compartmentation. (A) In serum, depletion of transamination substrates (Glu, Asp, Ser) only correlated with 2KG, whereas ketone body elevation was strongly linked to 2KIC. (B) In brain tissue, both 2KIC and 2KG showed strong positive correlations with Leu, 3HB, and AcAc and negative correlations with Glu, Asp, and GABA. Transamination substrates (Glu, Asp) were more closely linked to 2KG. 2KG, 2-ketoglutarate; 2KIC, 2-ketoisocaproate; 3HB, 3-hydroxybutyrate; AcAc, acetoacetate; Ala, alanine; Asp, aspartate; Cit, citrate; GABA, 4-aminobutyric acid; Lac, lactate; Gln, glutamine; Glu, glutamate; Leu, leucine; Pyr, pyruvate; Ser, serine, Suc, succinate.

In serum, 2KG also correlated inversely with serine (Figure 4A), likely signaling altered flux through hepatic phosphoserine aminotransferase 1 (PSAT1; see Equation S1, supplemental information). Glutamine decreased roughly 50% in brain (g = −3.1, q = 0.033) and 40% in serum (g = −5.3, q = 0.015). Although GABA levels were unchanged at P4, they correlated inversely with glutamate, 2KIC, and 2KG (Figure 4B). These results indicate that glutamate-ammonia ligase (GLUL; *astrocytes*) and glutamate decarboxylase 1 (GAD1; *neurons*) are vulnerable to substrate depletion under the influence of 2KIC (see Equations S2 and S3, supplemental information).

Untreated neonatal mice showed widespread reductions of circulating amino acids, including SLC7A5 transporter competitors whose cerebral uptake is subject to competitive inhibition by BCAAs (Table S1; Figure S2). In contrast, brain amino acids remained stable, widening their concentration gradients with blood 2- to 6-fold. Taurine was the only non-core amino acid significantly elevated in brain at P4 (+26%, q = 0.013, g = +8.0), possibly reflecting osmotic compensation or altered sulfur amino acid metabolism during intoxication.

2KIC correlated with the ketone bodies 3-hydroxybutyrate (3HB) and acetoacetate (AcAc) in both compartments (Figure 4), though elevations in brain (g = +1.3 and +1.9) and serum (g = +2.9 and +3.4) did not reach significance after global FDR correction. Molar ratios of 3HB/AcAc suggested divergent redox shifts between compartments: a ∼50% decrease in serum but nearly 3-fold increase in brain (Figure S3). Thus, elevated cerebral 2KIC triggers a complex neurochemical cascade characterized by disruption of transamination balances, neurotransmitter pools, ketone body homeostasis, and energy metabolism, with unique features in the brain as compared with peripheral tissues.

### Systemic dual-gene therapy restores cerebral metabolic homeostasis

To test whether systemic gene replacement could reverse neurochemical intoxication, we administered A-BiP-B (2.7 × 10^13^ vg/kg intravenously by facial vein) to newborn *Bckdha*−/− mice on P0. The A-BiP-B genome (4.1 kb) carried opti-h*BCKDHA* and opti-h*BCKDHB* under the control of a central bidirectional cytomegalovirus (CMV) enhancer with flanking dual chicken β-actin promoters and SV40/rβ-globin polyA signals, packed in AAV9 (Figure S1).^17^ All treated *Bckdha−/−* mice survived on unrestricted 20% protein chow until sacrifice at P140. We did not perform formal behavioral or neurological testing; accordingly, conclusions from this study are limited to biochemical and survival endpoints.

**Figure 5:**
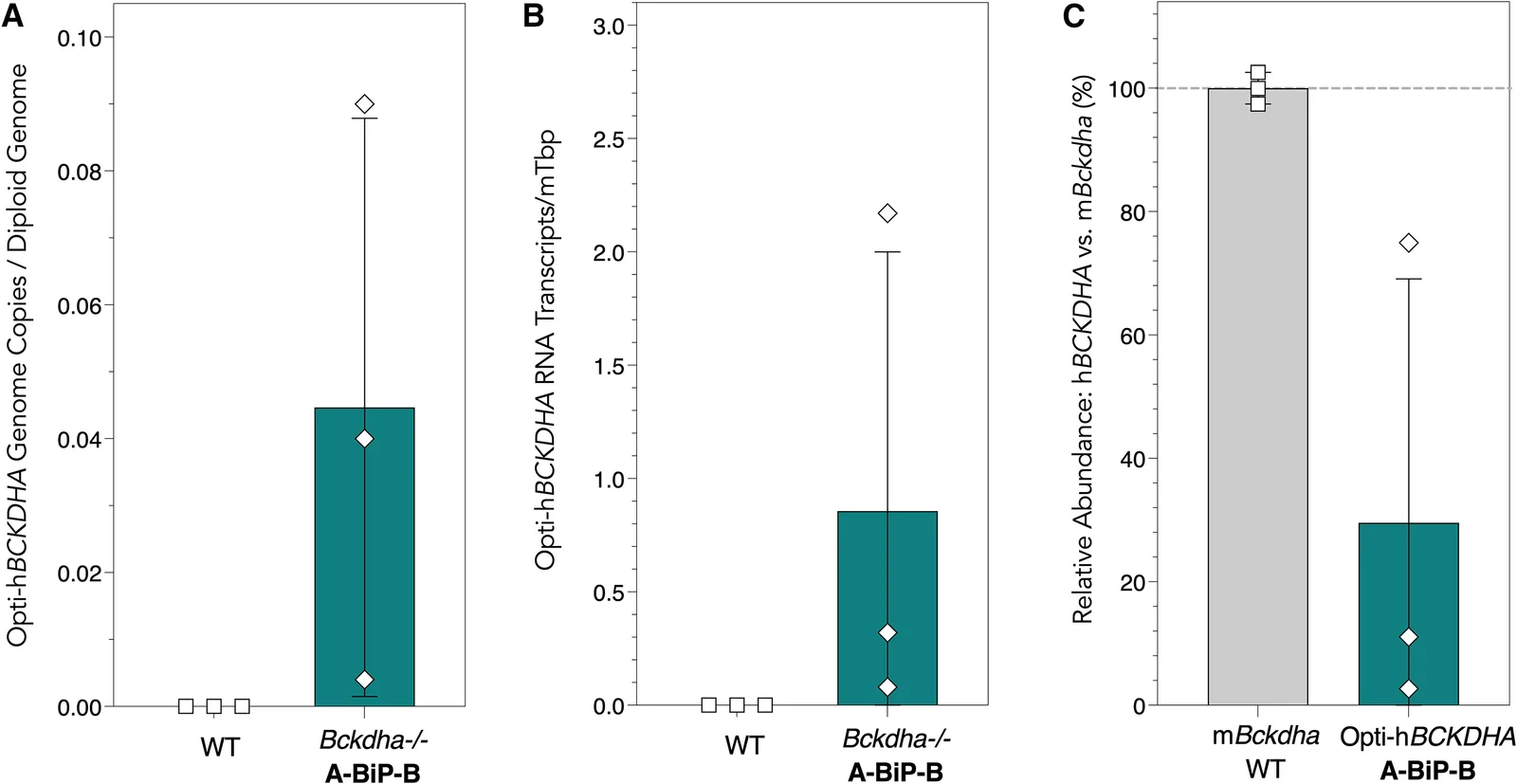
AAV9-A-BiP-B transgene expression in *Bckdha−/−* mouse brain tissue (A) We detected low levels of opti-h*BCKDHA* in brains of vector-treated P140 *Bckdha−/−* mice. (B) Opti-h*BCKDHA* mRNA produced from the transgene was only found in vector-treated (and not WT) mice. (C) Vector transgene in *Bckdha−/−* mouse brain produced relative mRNA abundance of 29.6% (median 11.1%; range: 2.7%–72.3%) as compared with endogenous *Bckdha* RNA in WT mouse brain. mTbp, TATA-binding protein-encoding mRNA; opti, codon-optimized; WT, wild-type.

Droplet digital PCR (QX200, Bio-Rad) confirmed vector delivery to the brain (Figure 5). Opti-h*BCKDHA* mRNA was expressed at roughly 30% of endogenous m*Bckdha* levels (normalized to *Tbp*), and A-BiP-B supported abundant co-expression of BCKDHA and BCKDHB proteins in brain and peripheral tissues.^17^

Vector-treated *Bckdha−/−* mice were protected from fatal neonatal encephalopathy. All core BCKDH–BCAT module metabolites in brain (BCAAs, BCKAs, 2KG, glutamate, and glutamine) returned to WT range, with no significant differences after global FDR correction (Table 2; Figure 1). Cerebral 2KIC (q = 0.85, g = −0.27), 2KG (q = 0.81, g = +0.39), and all three BCAAs were statistically indistinguishable from WT after global FDR correction, though valine (+64%, g = +4.57, q = 0.41) and isoleucine (+50%, g = +2.61, q = 0.41) showed residual elevations with effect sizes that exceeded the minimum detectable threshold for this design. Glutamate, glutamine, aspartate, GABA, and all secondary transamination and energy substrates similarly showed no significant differences from WT, nor did redox pairs or SLC7A5 competitor amino acids (Table 2). Of note, glutamine showed a residual 34% reduction that did not reach significance (q = 0.41, g = −2.0), a trend that may warrant evaluation in larger cohorts. The 3HB/AcAc molar ratio returned to the WT range (Figure S3). Alloisoleucine was undetectable in both treated and WT brain.

In contrast, serum BCKAs remained partially elevated in treated MSUD mice at P140, most notably 2KIC (56 vs. 14 μmol/L; q = 0.001, g = +16.8) and 2KIV (22 vs. 6 μmol/L; q = 0.020, g = +16.6; Table S2), representing incomplete peripheral metabolic correction. However, the residual concentration of 2KIC was roughly 50-fold lower than levels observed during neonatal intoxication (∼2,800 μmol/L) and—critically—brain tissue 2KIC returned to the WT range (85 vs. 87 μmol/g in WT; q = 0.85), well below the concentrations at which 2KIC reverses BCAT2 flux in astrocytes.^9^ In this model, partial restoration of CNS BCKDH activity was sufficient to stabilize cerebral neurochemistry despite residual peripheral biochemical abnormalities.

## Discussion

### BCKDH deficiency subverts a vital neurochemical network

**Figure 6:**
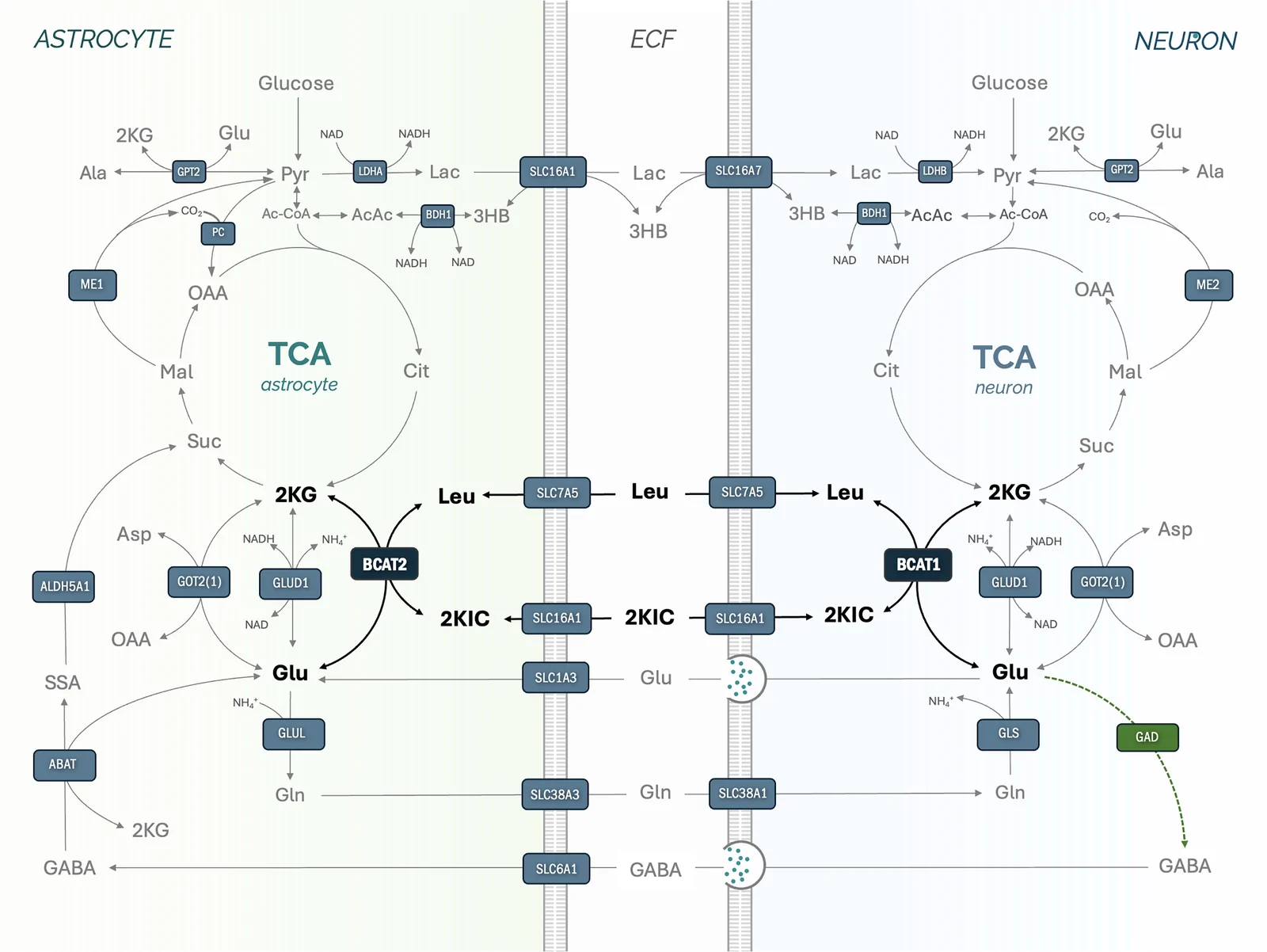
Integration of cerebral leucine, 2KIC, neurotransmitter, and energy metabolism Mitochondrial BCAT2 and cytosolic BCAT1 are enriched in astrocytes and neurons, respectively. BCAT forward and reverse reaction rates are governed by the law of mass action. High concentrations of 2KIC bias NH_2_ transfer from Glu to 2KG to yield leucine and alter the disposition of Glu. A 3-fold shift in the 2KG to Glu ratio produces secondary effects on multiple chemical processes, including the TCA cycle and synthesis of Asp, Ala, Gln, and GABA. 2KG, 2-ketoglutarate; 2KIC, 2-ketoisocaproate; 3HB, 3-hydroxybutyrate; AcAc, acetoacetate; Ac-CoA, acetyl-coenzyme A; Ala, alanine; Asp, aspartate; BCAT, branched-chain aminotransferase; Cit, citrate; CO_2_, carbon dioxide; ECF, extracellular fluid; GABA, 4-aminobutyric acid; Gln, glutamine; Glu, glutamate; Lac, lactate; Leu, leucine; Mal, malate; NAD(H), oxidized (and reduced) nicotinamide adenine dinucleotide; NH4^+^, ammonium ion; OAA, oxaloacetate; Pyr, pyruvate; SSA, succinic semialdehyde; Suc, succinate; TCA, tricarboxylic acid. Enzymes and transporters are abbreviated according to International Protein Nomenclature Guidelines (https://www.ncbi.nlm.nih.gov/genbank/internatprot_nomenguide/).

Cerebral leucine metabolism is intertwined with neurotransmitter synthesis and cerebral energy flux (Figure 6).^10^^,^^21^^,^^22^ Under physiological conditions, leucine enters the brain more rapidly than any other amino acid^6^ and is metabolized in astrocytes by BCAT2,^23^ transferring nitrogen to 2KG and generating a substantial fraction of cerebral glutamate.^10^ Glutamate thus formed is efficiently shuttled to neurons to support excitatory neurotransmission or fuel the TCA cycle.^22^ As the principal excitatory transmitter and substrate for its inhibitory counterpart (GABA), glutamate availability exerts strong control over synaptic signaling and cerebral energy demand.^24^^,^^25^^,^^26^ Most inherited disorders that perturb the glutamate-energy network cause severe encephalopathy early in life (Table S4). The intractable nature of these disorders underscores why the reversibility of MSUD intoxication matters mechanistically, not just descriptively.

### 2KIC drives acute intoxication

Targeted metabolomics confirmed that pathogenic elevations of 2KIC shift the glutamate-2KG equilibrium *in vivo*. At high concentrations, 2KIC reverses BCAT2 flux, diverting nitrogen from glutamate to leucine, elevating 2KG, and depleting glutamate. These mass-action effects propagate through linked transamination reactions, lowering aspartate, alanine, serine, and glutamine—metabolites whose steady-state concentrations depend on glutamate, 2KG, and/or their concentration ratio (Figure S4). The resulting neurochemical cascade disrupts both excitatory neurotransmission and cerebral energy metabolism while altering amino acid distribution between peripheral and central compartments.

2KIC intoxication also perturbs ketone body homeostasis. The divergent 3-hydroxybutyrate/acetoacetate (3HB/AcAc) ratios observed in serum versus brain likely reflect compartment-specific effects on redox balance and mitochondrial metabolism, potentially mediated by disruption of the malate-aspartate shuttle (Figure S5).^10^^,^^25^^,^^27^ Consistent with this interpretation, elevated tissue 2KG was correlated with higher concentrations of citrate and succinate. Prior work shows that 2KIC suppresses oxidative phosphorylation^27^^,^^28^^,^^29^ and inhibits 2KG dehydrogenase,^27^^,^^28^ providing a mechanistic explanation for the rapid yet reversible collapse of neurotransmitter and energy metabolism during MSUD crises.

Cerebral GABA tracked inversely with 2KIC, glutamate, and particularly 2KG across cohorts, reinforcing its dependence on the broader glutamate-energy network. GABA deficiency is a consistent feature of MSUD in both patients and animal models (Table S5).^18^^,^^30^^,^^31^ In neonatal *Bckdha−/−* mice, however, GABA levels were preserved despite marked glutamate depletion. This reflects developmental timing: cerebral GABA normally increases more than 10-fold between birth and maturity,^32^ which underscores the dynamic vulnerability of inhibitory neurotransmission across developmental stages.

### Current therapies cannot restore cerebral homeostasis

These findings highlight fundamental limitations of existing MSUD therapies. Dietary management provides only fragmentary peripheral metabolic control–not enough to protect the brain^4^–whereas liver transplantation establishes an effective peripheral sink for leucine and 2KIC but does not correct CNS metabolism.^5^ This disconnect likely explains why neuropsychiatric morbidity often persists in transplanted patients decades after durable normalization of circulating biomarkers.^5^^,^^33^

### Dual-gene therapy rescues the decisive CNS compartment

Systemic delivery of A-BiP-B restored cerebral h*BCKDHA* mRNA expression and returned brain amino acid, neurotransmitter, and organic acid profiles to the WT range (Table 2). All core BCKDH-BCAT module metabolites—including 2KIC, 2KG, glutamate, and glutamine—were statistically indistinguishable from WT after global FDR correction, with effect sizes near zero (Table 2). Serum BCKAs remained partially elevated at P140, most notably 2KIC (56 vs. 14 μmol/L in WT), representing incomplete peripheral correction. However, this residual concentration is roughly 50-fold lower than neonatal crisis levels (∼2,800 μmol/L) and falls below the threshold at which 2KIC reverses BCAT2 flux in astrocytes.^9^ Critically, brain tissue 2KIC returned to the WT range (85 vs. 87 μmol/g; q = 0.85), confirming that tissue concentrations—not circulating levels—are mechanistically decisive for the glutamate-2KG equilibrium. In this respect, our findings invert the transplant paradigm: whereas CNS-restricted BCKDH deficiency causes neurochemical dysfunction despite normal plasma biomarkers,^5^^,^^12^ partial CNS correction preserves cerebral homeostasis even in the setting of residual peripheral biochemical abnormalities.

### Cross-correction and cellular mechanisms

The normalization of brain neurochemistry observed here likely reflects both cell-autonomous and non-autonomous mechanisms. Neonatal systemic AAV9 transduces neurons and astrocytes broadly,^13^^,^^14^^,^^34^^,^^35^ and opti-h*BCKDHA* mRNA was detectable in brain at roughly 30% of endogenous levels (Figure 5). Because cerebral leucine metabolism is distributed across cell types—BCAT2 predominates in astrocytes, BCAT1 in neurons, and glutamate-glutamine cycling operates between them^22^^,^^36^—restoration of BCKDH activity in even a fraction of metabolically strategic cells may be sufficient to stabilize tissue-level mass-action equilibria. Specifically, correcting BCKDH in some astrocytes could normalize the BCAT2-dependent glutamate-2KIC exchange that initiates the intoxication cascade, with downstream benefits propagated to neurons through established metabolic coupling.^9^^,^^26^ The present dataset does not distinguish between cell-autonomous correction and metabolic cross-correction between transduced and untransduced cells; resolving this question—e.g., through cell-type-specific conditional rescue—is an important direction for future investigation.

### Limitations

This study was designed as a paired serum-brain metabolomics analysis in a neonatal-lethal model, prioritizing depth of biochemical phenotyping within each compartment. The cohort size (*n* = 3 per group) is small. We therefore report exact *n*, standard deviation (SD), q values, and Hedges’ g for all analytes. For the primary neurochemical endpoints, effect sizes were large (Hedges’ g = 3–17), indicating strong separation of group distributions for core BCKDH-BCAT module metabolites despite the limited sample size. Secondary endpoints, e.g., transamination substrates and the residual glutamine reduction observed at P140 (−34%, g = −2.0, q = 0.41), fell below the discovery threshold and warrant confirmation in larger cohorts.

We restricted the study to female mice to reduce biological variance and maintain consistency with our prior metabolomics pipeline. Sex differences in AAV9 transduction efficiency and neurometabolite baselines have been described in other contexts,^37^^,^^38^ and they could influence both the intoxication phenotype and the therapeutic response. Ongoing studies include both sexes to evaluate generalizability.

This study reports biochemical and survival endpoints. Treated mice survived on unrestricted diet with no overt behavioral abnormalities, but we did not perform formal neurobehavioral, electrophysiological, or cognitive assessments. Whether the neurochemical rescue demonstrated here translates into durable functional improvement remains to be established through dedicated behavioral studies.

### Translational implications

In MSUD, a single enzymatic defect destabilizes a highly interconnected neurotransmitter-energy network that sustains normal brain function, making neurochemical homeostasis the appropriate therapeutic benchmark. The present data establish that systemic dual-gene therapy can restore this homeostasis within the CNS while enabling survival on unrestricted protein intake. This compartment-specific efficacy signal informs rational preclinical development. Dose optimization, durability, biodistribution, immunogenicity, and long-term safety studies remain necessary before clinical translation and are in progress. More broadly, this compartment-focused framework may apply to other metabolic encephalopathies in which peripheral biochemical control fails to predict neurological risk (Table S4).

## Materials and methods

### Animal procedures

All animal procedures were reviewed and approved by the Institutional Animal Care and Use Committee at UMass Chan Medical School (PROTO202000184) and complied with ethical regulations. *Bckdha*−/− mice were recovered from a cryopreserved line (Canadian Mouse Mutant Repository) carrying an intron-exon disruption. A heterozygous colony was generated by in vitro fertilization and bred to homozygosity.

### Experimental design

We studied four groups (*n* = 3 female mice per group): P4 WT vs. P4 MSUD, and P140 WT vs. P140 MSUD treated. WT controls were age-matched because several neurochemicals vary with age. Mice were co-housed across groups; no animals were excluded. Cohorts were restricted to female mice to reduce sex-related variance in this proof-of-concept metabolomic study and to maintain consistency with our prior analytical pipeline. Sex-specific effects on AAV9 transduction and neurometabolite baselines were not evaluated and remain a limitation (see discussion). Brains were collected after PBS perfusion, dissected, snap-frozen, and stored at −80°C. Serum and brain were harvested the same day.

### Vector design and delivery

The therapeutic vector was built on an ssAAV2-ITR backbone (4.1 kb) encoding opti-h*BCKDHA* and opti**-**h*BCKDHB* in a bidirectional cassette driven by a CMV enhancer with dual chicken β-actin promoters and terminated with SV40 and rabbit β-globin polyA signals. The DNA cassette was packaged in recombinant AAV9 formulated in buffered aqueous suspension. Newborn *Bckdha*−/− mice received 2.7 × 10^13^ vg/kg intravenously by facial vein on P0 and remained on chow containing 20% protein.

### Nucleic acid assays

DNA and RNA were extracted from brain. Droplet digital PCR (QX200, Bio-Rad) was performed with TaqMan assays targeting opti-h*BCKDHA*, endogenous m*Bckdha*, and *Tbp*. RNA was reverse-transcribed, and relative expression of opti-h*BCKDHA* was normalized to *Tbp* and endogenous m*Bckdha*. Full primer and probe sequences are listed in Method S1.

### Metabolite quantitation

Amino acids were quantified by liquid chromatography-tandem mass spectrometry; organic acids were analyzed by gas chromatography-mass spectrometry operated in selected ion monitoring mode with positive electron ionization. Stable-isotope internal standards were added to serum and brain homogenates prior to analysis. Serum analytes are reported in μmol/L, brain amino acids in nmol/g tissue, and organic acids in μmol/g tissue.

For biological interpretation, metabolites are organized into four biochemical families: (1) the core BCKDH–BCAT module (BCAAs, BCKAs, 2KG, glutamate, and glutamine), representing the primary enzymatic defect and its proximate mass-action consequences; (2) transamination and energy substrates (aspartate, alanine, serine, GABA, and TCA cycle intermediates), reflecting downstream equilibrium shifts biochemically close to shifts in the 2KG/glutamate equilibrium; (3) cytosolic and mitochondrial redox pairs (lactate/pyruvate, 3HB/AcAc); and (4) amino acid substrates (histidine, methionine, phenylalanine, threonine, tryptophan, and tyrosine) that enter the brain via the shared large neutral amino acid transporter (SLC7A5) and are therefore subject to competitive transport inhibition by BCAAs. This organizational scheme serves biological readability but does not influence statistical inference, which was performed globally across all observations for maximal stringency (see below).

### Targeted mass-action analysis

Metabolite data were analyzed according to the law of mass action.^20^ Chemical equilibria for BCAT2, GOT2, and GPT2 are included in the main text (Equations 1, 2, 3, and 4). PSAT1, GLUL, and GAD1 equilibria are detailed in the Method S1 (Equations S1–S3). Fits used linear or one-phase decay models depending on data shape; goodness of fit was reported as R^2^ (and Sy.x when relevant). Correlations are computed across the full experimental range and may partly reflect between-group separation; within-cohort analyses and Spearman ρ are provided in supplemental information.

### Statistics

Group comparisons were performed using unpaired, two-tailed Welch’s t tests (assuming independent variances) in Prism 10 (GraphPad). To maximize stringency, *p* values were corrected for multiple comparisons across all analytes within each age x compartment comparison (27 metabolites per comparison) using the two-stage linear step-up method of Benjamini, Krieger, and Yekutieli (FDR set at 5%). The resulting q values reflect the most conservative correction applied to the full analyte panel, without parsing by metabolite family. A q value ≤0.05 was considered a discovery.

Effect sizes are reported as Hedges’ g, the bias-corrected standardized mean difference appropriate for small samples. For *n* = 3 per group, the correction factor J ≈ 0.80 attenuates the upward bias inherent in Cohen’s d. Given the small cohort size, we report Hedges’ g to allow direct assessment of effect magnitude. For reference, the minimum detectable effect size at 80% power (two-sided α = 0.05) for this design is approximately |g| = 2.5. Metabolite ratios were log-transformed prior to testing.

Data are reported as mean ± SEM. SDs are provided in tables to allow independent assessment of within-group variability. Some figures use Z scores for visual clarity; gray bands indicate ±2 and ±3 SD thresholds relative to age-matched WT controls. Significance is annotated as ∗q ≤ 0.05, ∗∗q ≤ 0.01, or ∗∗∗q ≤ 0.001.

## Data and code availability

Datasets generated and analyzed during the current study are available from the corresponding author upon reasonable request. Raw metabolomic data, summary statistics, and analysis scripts supporting the findings of this study will be provided to qualified researchers for non-commercial research purposes, consistent with institutional and ethical guidelines.

## Acknowledgments

The Wang Lab is supported by grant from the 10.13039/100000002National Institutes of Health (NIH) (P01HL158506). The Gao Lab is supported by grants from the 10.13039/100000002NIH (R01NS076991-01, P01AI100263-01, P01HL131471–02, R01AI121135, UG3HL147367–01, R01HL097088, and U19AI149646-01) and Cystic Fibrosis Foundation. The Gray-Edwards Lab received funding from the 10.13039/100003936MSUD Family Support Group. The authors thank members of the AAV vector core at University of Massachusetts Chan Medical School who produced AAV9-A-BiP-B for murine experiments. We are grateful to the many patients and families who continue to inspire and support our research efforts.

## Author contributions

Conceptualization: H.G.-E., G.G., S.T., D.W., K.A.S.; methodology: J.W., C.T.T., P.R.L., S.T., K.A.S.; investigation: J.W., C.T.T., P.R.L., S.T., K.A.S.; data curation: J.W., C.T.T., P.R.L., S.T., K.A.S.; visualization: K.A.S.; funding acquisition: G.G., S.T., D.W.; project administration: S.T., D.W., K.A.S.; supervision: G.G., S.T., D.W., K.A.S.; writing – original draft: J.W., C.T.T., P.R.L., K.A.S.; writing – review and editing: J.W., C.T.T., P.R.L., H.G.-E., G.G., S.T., D.W., K.A.S.

## Declaration of interests

This work was funded in part through a sponsored research agreement with ASC Therapeutics (Milpitas, California). None of the authors received personal compensation from ASC Therapeutics or hold financial interest in the company. J.W., G.G., K.A.S., and D.W. are inventors of a patent application filed by University of Massachusetts Chan Medical School concerning the work described in this study (WO2020210595 - AAV-mediated gene therapy for maple syrup urine disease (MSUD)). G.G. is a scientific co-founder of Voyager Therapeutics, Adrenas Therapeutics, and Aspa Therapeutics and holds equity in the companies. K.A.S. is the founder of Plowshare Therapies LLC and holds equity in this company.

## Supplemental information

Document S1. Figures S1–S5, Tables S1–S6, and Method S1

Document S2. Article plus supplemental information

## References

[1] Chuang D.T., Shih V.E., Scriver C.R., Beaudet A.L., Valle D., Sly W.S. (2001). The Metabolic and Molecular Bases of Inherited Disease.

[2] Strauss K.A., Puffenberger E.G., Morton D.H. (2009). Maple Syrup Urine Disease. http://www.ncbi.nlm.nih.gov/bookshelf/br.fcgi?book=gene&part=msud.

[3] Menkes J.H., Hurst P.L., Craig J.M. (1954). A new syndrome: progressive familial infantile cerebral dysfunction associated with an unusual urinary substance. Pediatrics.

[4] Strauss K.A., Carson V.J., Soltys K., Young M.E., Bowser L.E., Puffenberger E.G., Brigatti K.W., Williams K.B., Robinson D.L., Hendrickson C. (2020). Branched-chain alpha-ketoacid dehydrogenase deficiency (maple syrup urine disease): Treatment, biomarkers, and outcomes. Mol. Genet. Metab..

[5] Muelly E.R., Moore G.J., Bunce S.C., Mack J., Bigler D.C., Morton D.H., Strauss K.A. (2013). Biochemical correlates of neuropsychiatric illness in maple syrup urine disease. J. Clin. Invest..

[6] Smith Q.R., Stoll J.S., Pardridge W.M. (1998). Introduction to the Blood-Brain Barrier.

[7] Kamei A., Takashima S., Chan F., Becker L.E. (1992). Abnormal dendritic development in maple syrup urine disease. Pediatr. Neurol..

[8] Araújo P., Wassermann G.F., Tallini K., Furlanetto V., Vargas C.R., Wannmacher C.M., Dutra-Filho C.S., Wyse A.T., Wajner M. (2001). Reduction of large neutral amino acid levels in plasma and brain of hyperleucinemic rats. Neurochem. Int..

[9] Yudkoff M., Daikhin Y., Nissim I., Pleasure D., Stern J., Nissim I. (1994). Inhibition of astrocyte glutamine production by alpha-ketoisocaproic acid. J. Neurochem..

[10] Yudkoff M., Daikhin Y., Nissim I., Horyn O., Luhovyy B., Luhovyy B., Lazarow A., Nissim I. (2005). Brain amino acid requirements and toxicity: the example of leucine. J. Nutr..

[11] Knerr I., Colombo R., Urquhart J., Morais A., Merinero B., Oyarzabal A., Pérez B., Jones S.A., Perveen R., Preece M.A. (2019). Expanding the genetic and phenotypic spectrum of branched-chain amino acid transferase 2 deficiency. J. Inherit. Metab. Dis..

[12] Kuhs A.C., Ohl L., Thurston T., Singh J., Bhuyan S., Grandinette S., Xu J., Siemsgluess S.A., Jakher Y., Ahrens-Nicklas R.C. (2025). Contribution of Brain Intrinsic Branched-Chain Amino Acid Metabolism in a Novel Mouse Model of Maple Syrup Urine Disease. J. Inherit. Metab. Dis..

[13] Foust K.D., Nurre E., Montgomery C.L., Hernandez A., Chan C.M., Kaspar B.K. (2009). Intravascular AAV9 preferentially targets neonatal neurons and adult astrocytes. Nat. Biotechnol..

[14] Zincarelli C., Soltys S., Rengo G., Rabinowitz J.E. (2008). Analysis of AAV serotypes 1-9 mediated gene expression and tropism in mice after systemic injection. Mol. Ther..

[15] Pontoizeau C., Simon-Sola M., Gaborit C., Nguyen V., Rotaru I., Tual N., Colella P., Girard M., Biferi M.G., Arnoux J.B. (2022). Neonatal gene therapy achieves sustained disease rescue of maple syrup urine disease in mice. Nat. Commun..

[16] Pontoizeau C., Gaborit C., Tual N., Simon-Sola M., Rotaru I., Benoist M., Colella P., Lamazière A., Brassier A., Arnoux J.B. (2024). Successful treatment of severe MSUD in Bckdhb(-/-) mice with neonatal AAV gene therapy. J. Inherit. Metab. Dis..

[17] Wang J., Poskitt L.E., Gallagher J., Puffenberger E.G., Wynn R.M., Shishodia G., Chuang D.T., Beever J., Hardin D.L., Brigatti K.W. (2025). BCKDHA-BCKDHB digenic gene therapy restores metabolic homeostasis in two mouse models and a calf with classic maple syrup urine disease. Sci. Transl. Med..

[18] Prensky A.L., Moser H.W. (1966). Brain lipids, proteolipids, and free amino acids in maple syrup urine disease. J. Neurochem..

[19] Peerboom C., Wierenga C.J. (2021). The postnatal GABA shift: A developmental perspective. Neurosci. Biobehav. Rev..

[20] Ferner R.E., Aronson J.K. (2016). Cato Guldberg and Peter Waage, the history of the Law of Mass Action, and its relevance to clinical pharmacology. Br. J. Clin. Pharmacol..

[21] Hutson S.M., Lieth E., LaNoue K.F. (2001). Function of leucine in excitatory neurotransmitter metabolism in the central nervous system. J. Nutr..

[22] McKenna M.C. (2007). The glutamate-glutamine cycle is not stoichiometric: fates of glutamate in brain. J. Neurosci. Res..

[23] Yudkoff M., Daikhin Y., Grunstein L., Nissim I., Stern J., Pleasure D., Nissim I. (1996). Astrocyte leucine metabolism: significance of branched-chain amino acid transamination. J. Neurochem..

[24] Magistretti P.J., Pellerin L., Rothman D.L., Shulman R.G. (1999). Energy on demand. Science.

[25] Rothman D.L., Behar K.L., Dienel G.A. (2024). Mechanistic stoichiometric relationship between the rates of neurotransmission and neuronal glucose oxidation: Reevaluation of and alternatives to the pseudo-malate-aspartate shuttle model. J. Neurochem..

[26] Sibson N.R., Shen J., Mason G.F., Rothman D.L., Behar K.L., Shulman R.G. (1998). Functional energy metabolism: in vivo 13C-NMR spectroscopy evidence for coupling of cerebral glucose consumption and glutamatergic neuronalactivity. Dev. Neurosci..

[27] Zemniaçak Â.B., Ribeiro R.T., das Neves G.M., Cunha S.d.A., Tavares T.Q., Carvalho A.V.S., Netto C.A., Castilho R.F., Wajner M., Amaral A.U. (2025). alpha-Ketoisocaproic Acid Disrupts Mitochondrial Bioenergetics in the Brain of Neonate Rats: Molecular Modeling Studies of alpha-ketoglutarate Dehydrogenase Subunits Inhibition. Neurochem. Res..

[28] Amaral A.U., Wajner M. (2022). Pathophysiology of maple syrup urine disease: Focus on the neurotoxic role of the accumulated branched-chain amino acids and branched-chain alpha-keto acids. Neurochem. Int..

[29] Farias H.R., Gabriel J.R., Cecconi M.L., Lemos I.S., de Rezende V.L., Wessler L.B., Duarte M.B., Scaini G., de Oliveira J., Streck E.L. (2021). The metabolic effect of alpha-ketoisocaproic acid: in vivo and in vitro studies. Metab. Brain Dis..

[30] Dodd P.R., Williams S.H., Gundlach A.L., Harper P.A., Healy P.J., Dennis J.A., Johnston G.A. (1992). Glutamate and gamma-aminobutyric acid neurotransmitter systems in the acute phase of maple syrup urine disease and citrullinemia encephalopathies in newborn calves. J. Neurochem..

[31] Zinnanti W.J., Lazovic J., Griffin K., Skvorak K.J., Paul H.S., Homanics G.E., Bewley M.C., Cheng K.C., Lanoue K.F., Flanagan J.M. (2009). Dual mechanism of brain injury and novel treatment strategy in maple syrup urine disease. Brain..

[32] Chen S., Lee J., Truong T.M., Alhassen S., Baldi P., Alachkar A. (2021). Age-Related Neurometabolomic Signature of Mouse Brain. ACS Chem. Neurosci..

[33] Shellmer D.A., DeVito Dabbs A., Dew M.A., Noll R.B., Feldman H., Strauss K.A., Morton D.H., Vockley J., Mazariegos G.V. (2011). Cognitive and adaptive functioning after liver transplantation for maple syrup urine disease: a case series. Pediatr. Transpl..

[34] Gray S.J., Matagne V., Bachaboina L., Yadav S., Ojeda S.R., Samulski R.J. (2011). Preclinical differences of intravascular AAV9 delivery to neurons and glia: a comparative study of adult mice and nonhuman primates. Mol. Ther..

[35] Dashkoff J., Lerner E.P., Truong N., Klickstein J.A., Fan Z., Mu D., Maguire C.A., Hyman B.T., Hudry E. (2016). Tailored transgene expression to specific cell types in the central nervous system after peripheral injection with AAV9. Mol. Ther. Methods Clin. Dev..

[36] Hutson S.M., Berkich D., Drown P., Xu B., Aschner M., LaNoue K.F. (1998). Role of branched-chain aminotransferase isoenzymes and gabapentin in neurotransmitter metabolism. J. Neurochem..

[37] Abdelhamid L., Mazor R. (2025). Can Sex-based Variations in the Immune Responses to AAV Gene Therapy Affect Safety and Efficacy? A Review of Current Understanding. AAPS J..

[38] Davidoff A.M., Ng C.Y.C., Zhou J., Spence Y., Nathwani A.C. (2003). Sex significantly influences transduction of murine liver by recombinant adeno-associated viral vectors through an androgen-dependent pathway. Blood.

