# Supplementary material for: Systemic dual-gene therapy reverses biochemical intoxication in the central metabolic compartment of Bckdha−/− mice
Source: Mol Ther. 2026 May 13;34(8):4569–80. doi: 10.1016/j.ymthe.2026.05.008 (PMC13464135; doi:10.1016/j.ymthe.2026.05.008)
Supplement: Supplementary file 1 [file mmc1.pdf]

YMTHE, Volume *34*

Supplemental Information

Systemic dual-gene therapy reverses biochemical intoxication in the central metabolic compartment of *Bckdha*−/− mice

Jiaming Wang, Coleman T. Turgeon, Perry R. Loken, Heather Gray-Edwards, Guangping Gao, Silvia Tortorelli, Dan Wang, and Kevin A. Strauss

Table S1. Serum Metabolites in Wild Type and *Bckdha-/-* Mice, Postnatal Day 4: Untreated

		Wild Type (n=3)			MSUD (n=3)				Statistics			
**Metabolite**	**Units**	**Mean**	**SD**	**SEM**	**Mean**	**SD**	**SEM**	**Δ%**	**p value**	**q value**	**Discovery***	**Hedges' g**
**Core BCKDH–BCAT Module**												
Leucine	µmol/L	**305**	21.1	12.2	**4518**	239	138	1380	0.0010	**0.0054**	**Yes**	19.84
Isoleucine	µmol/L	**255**	14.0	8.1	**1527**	97.2	56.1	500	0.0016	**0.0073**	**Yes**	14.66
Valine	µmol/L	**488**	23.1	13.3	**2202**	70.7	40.8	351	0.0002	**0.0024**	**Yes**	26.06
2-Ketoisocaproate	µmol/L	**27.8**	4.1	2.4	**2813**	364	210	10029	0.0056	**0.0152**	**Yes**	8.67
2-Keto-3-methyl-N-valerate	µmol/L	**41.2**	10.1	5.8	**853**	121	69.7	1968	0.0070	**0.0172**	**Yes**	7.58
2-Ketoisovalerate	µmol/L	**12.9**	1.0	0.60	**655**	55.9	32.3	4964	0.0025	**0.0097**	**Yes**	12.99
2-Ketoglutarate	µmol/L	**11.4**	3.2	1.9	**17.9**	1.9	1.1	57	0.0529	0.0714	No	1.95
Glutamate	µmol/L	**198**	42.5	24.6	**127**	5.3	3.1	-36	0.0995	0.1279	No	-1.87
Glutamine	µmol/L	**934**	71.9	41.5	**565**	31.6	18.3	-40	0.0053	**0.0152**	**Yes**	-5.32
**Transamination and Energy Substrates**												
Aspartate	µmol/L	**48.3**	0.58	0.33	**19.3**	3.2	1.9	-60	0.0032	**0.0109**	**Yes**	-10.05
Alanine	µmol/L	**792**	52.5	30.3	**208**	42.5	24.5	-74	0.0002	**0.0024**	**Yes**	-9.78
Serine	µmol/L	**238**	60.3	34.8	**177**	20.3	11.7	-26	0.2157	0.2532	No	-1.08
Citrate	µmol/L	**206**	27.2	15.7	**379**	49.3	28.5	84	0.0117	**0.0242**	**Yes**	3.49
Succinate	µmol/L	**14.1**	1.4	0.80	**9.8**	1.3	0.74	-31	0.0165	**0.0300**	**Yes**	-2.61
Fumarate	µmol/L	**4.3**	0.64	0.37	**4.5**	0.53	0.30	3	0.7893	0.7893	No	0.19
Malate	µmol/L	**17.8**	3.4	2.0	**18.9**	0.84	0.48	6	0.6554	0.6806	No	0.33
**Redox Pairs**												
Lactate	µmol/L	**2195**	195	112	**1762**	302	174	-20	0.1173	0.1440	No	-1.36
Pyruvate	µmol/L	**38.1**	9.2	5.3	**33.2**	6.8	3.9	-13	0.4991	0.5390	No	-0.49
3-Hydroxybutyrate	µmol/L	**880**	165	95.2	**2553**	636	367	190	0.0378	0.0567	No	2.88
Acetoacetate	µmol/L	**337**	53.0	30.6	**1777**	473	273	428	0.0327	0.0519	No	3.43
**SLC7A5 Transporter Competitors**												
Histidine	µmol/L	**232**	24.8	14.3	**130**	2.9	1.7	-44	0.0178	**0.0300**	**Yes**	-4.65
Methionine	µmol/L	**126**	16.5	9.5	**74.7**	8.5	4.9	-41	0.0175	**0.0300**	**Yes**	-3.13
Phenylalanine	µmol/L	**103**	18.2	10.5	**113**	6.1	3.5	10	0.4348	0.4892	No	0.61
Threonine	µmol/L	**479**	53.5	30.9	**371**	39.4	22.7	-23	0.0521	0.0714	No	-1.85
Tryptophan	µmol/L	**101**	11.5	6.7	**44.3**	3.5	2.0	-56	0.0088	**0.0197**	Yes	-5.32
Tyrosine	µmol/L	**258**	14.6	8.5	**151**	11.0	6.4	-41	0.0008	**0.0054**	Yes	-6.57
**Other Relevant Metabolites**												
Alloisoleucine	µmol/L	**nd**			**295**	39.9	23.0		na			
Taurine	µmol/L	**933**	20.3	11.7	**579**	35.1	20.3	-38	0.0004	**0.0039**	Yes	-9.89

*Multiple, unpaired, two-tailed t tests with Welch correction (assuming individual variances), False Discovery Rate (FDR) set at 5%. The q value represents a p value corrected for multiple comparisons.

Abbreviations: BCAT, branched-chain amino acid transaminase; BCKDH, branched-chain 2-ketoacid dehydrogenase complex; GABA, 4-aminobutyric acid; MSUD, maple syrup urine disease; na, not applicable; nd, not detected; SD, standard deviation; SEM, standard error of the mean; Δ%, percent difference from wild type.

Table S2. Serum Metabolites in Wild Type and *Bckdha-/-* Mice, Postnatal Day 140: Treated with AAV9-A-BiP-B

		Wild Type (n=3)			MSUD (n=3)				Statistics			
**Metabolite**	**Units**	**Mean**	**SD**	**SEM**	**Mean**	**SD**	**SEM**	**Δ%**	**p value**	**q value**	**Discovery***	**Hedges' g**
**Core BCKDH–BCAT Module**												
Leucine	µmol/L	**261**	31.4	18.1	**345**	48.4	28.0	32	0.0750	0.3066	No	1.65
Isoleucine	µmol/L	**159**	20.3	11.7	**189**	32.9	19.0	19	0.2588	0.4368	No	0.89
Valine	µmol/L	**293**	29.5	17.0	**368**	42.9	24.8	26	0.0744	0.3066	No	1.64
2-Ketoisocaproate	µmol/L	**13.9**	1.6	0.90	**56.3**	2.4	1.4	305	0.0000	**0.0013**	**Yes**	16.75
2-Keto-3-methyl-N-valerate	µmol/L	**7.3**	0.58	0.33	**26.5**	3.5	2.0	262	0.0094	0.0850	No	6.11
2-Ketoisovalerate	µmol/L	**5.9**	0.06	0.04	**22.4**	1.1	0.65	281	0.0015	**0.0202**	**Yes**	16.60
2-Ketoglutarate	µmol/L	**28.8**	7.9	4.5	**39.9**	8.5	4.9	38	0.1746	0.4042	No	1.08
Glutamate	µmol/L	**78.7**	10.1	5.8	**61.0**	10.5	6.1	-22	0.1044	0.3186	No	-1.37
Glutamine	µmol/L	**817**	131	75.8	**697**	42.4	24.5	-15	0.2500	0.4368	No	-0.98
**Transamination and Energy Substrates**												
Aspartate	µmol/L	**11.0**	5.2	3.0	**5.0**	1.0	0.58	-55	0.1797	0.4042	No	-1.28
Alanine	µmol/L	**491**	79.4	45.8	**456**	42.2	24.3	-7	0.5406	0.6929	No	-0.45
Serine	µmol/L	**117**	20.4	11.8	**110**	12.9	7.4	-6	0.6316	0.7105	No	-0.34
Citrate	µmol/L	**283**	5.0	2.9	**266**	16.4	9.5	-6	0.2069	0.4298	No	-1.13
Succinate	µmol/L	**406**	64.6	37.3	**351**	154	89.0	-14	0.6123	0.7105	No	-0.37
Fumarate	µmol/L	**23.2**	4.8	2.8	**18.7**	2.3	1.3	-19	0.2417	0.4368	No	-0.96
Malate	µmol/L	**98.0**	21.7	12.5	**80.2**	9.5	5.5	-18	0.2926	0.4647	No	-0.85
**Redox Pairs**												
Lactate	µmol/L	**13614**	1062	613	**13858**	505	292	2	0.7443	0.7729	No	0.23
Pyruvate	µmol/L	**271**	70.3	40.6	**242**	35.8	20.7	-11	0.5646	0.6929	No	-0.42
3-Hydroxybutyrate	µmol/L	**214**	13.5	7.8	**149**	45.9	26.5	-30	0.1233	0.3330	No	-1.54
Acetoacetate	µmol/L	**3.4**	0.55	0.32	**2.8**	0.82	0.47	-18	0.3409	0.5113	No	-0.72
**SLC7A5 Transporter Competitors**												
Histidine	µmol/L	**89.7**	10.4	6.0	**82.7**	6.0	3.5	-8	0.3834	0.5448	No	-0.66
Methionine	µmol/L	**76.3**	7.8	4.5	**69.7**	14.3	8.3	-9	0.5277	0.6929	No	-0.46
Phenylalanine	µmol/L	**122**	7.1	4.1	**102**	13.2	7.6	-16	0.1062	0.3186	No	-1.48
Threonine	µmol/L	**155**	37.5	21.6	**149**	16.5	9.5	-4	0.8273	0.8273	No	-0.16
Tryptophan	µmol/L	**114**	12.9	7.4	**92.3**	8.7	5.0	-19	0.0795	0.3066	No	-1.60
Tyrosine	µmol/L	**95.0**	6.9	4.0	**73.0**	12.1	7.0	-23	0.0675	0.3066	No	-1.78
**Other Relevant Metabolites**												
Alloisoleucine	µmol/L	**nd**			**nd**				na			
Taurine	µmol/L	**806**	71.5	41.3	**786**	63.7	36.8	-3	0.7320	0.7729	No	-0.24

*Multiple, unpaired, two-tailed t tests with Welch correction (assuming individual variances), False Discovery Rate (FDR) set at 5%. The q value represents a p value corrected for multiple comparisons.

Abbreviations: BCAT, branched-chain amino acid transaminase; BCKDH, branched-chain 2-ketoacid dehydrogenase complex; GABA, 4-aminobutyric acid; MSUD, maple syrup urine disease; na, not applicable; nd, not detected; SD, standard deviation; SEM, standard error of the mean; Δ%, percent difference from wild type.

Table S3. Fate of ^{13}C-Glutamate in Cortical Astrocytes[a]

	Extracellular Glutamate (mM)		
	0.1	0.2	0.5
**Percentage of ^{13}C Label**			
Glutamine formation (^{13}C)	85	67	57
Metabolized via TCA cycle[b]	15	33	43
• Aspartate (^{13}C)	*nd*	5	14
• Lactate (1,2,3-^{13}C)	*nd*	21	15
• Glutamate (1,2,3-^{13}C)	15	7	14

[a] Data from McKenna et al., *J Neurochem* 66:386-393 (1996)[1].

[b] Percentage of labeled glutamate metabolized via the TCA cycle is represented by labeled ^{13}C-aspartate as well as lactate and glutamate labeled at carbons 1, 2, and 3.

Abbreviations: *nd*, not detected; TCA, tricarboxylic acid

Table S4. Genetic defects associated with moderate or severe psychomotor disability

Gene Symbol	Protein Name	Inheritance	OMIM	PubMed ID
BCAT2	branched-chain amino transferase 2 (mitochondrial)	AR	618850	31177572
GOT2	glutamate oxaloacetate transaminase 2	AR	618721	31422819
GPT2	glutamate pyruvate transaminase 2	AR	616281	25758935
PSAT1	phosphoserine aminotransferase 1	AR	610992	17436247
GLS	glutaminase	AR	618328	30575854
GLUL	glutamine synthetase	AR	610015	16267323
SLC38A3	glutamine transporter (SNAT3)	AR	619881	34605855
ABAT	GABA-transaminase	AR	613163	28411234
ALDH5A1	succinic semialdehyde dehydrogenase	AR	271980	22722631
PC	pyruvate carboxylase	AR	266150	6434849
SLC16A1	monocarboxylate transporter 1 (MCT1)	AR	616095	25390740
SLC25A12	aspartate-glutamate carrier 1	AR	612949	19641205
MDH1	malate dehydrogenase 1 (cytosolic)	AR	618959	31538237
MDH2	malate dehydrogenase 2 (mitochondrial)	AR	617339	27989324

Abbreviations: AR, autosomal recessive; ID, identification number; OMIM, Online Mendelian Inheritance in Man.

Table S5. Brain Metabolite Content as a Percentage in Comparison to Normal Tissue

	2KIC	2KG	Leucine	Glutamate	GABA	Aspartate	
Human post-mortem, DOL 25			456%	30%	19%	*	Prensky, Moser, 1966 [2]
Bovine classic MSUD (*BCKDHA* c.248C>T), P3-10*			1000%	40%	50%	20%	Dodd *et al*., 1992 [3]
Murine intermediate MSUD*,* P42			1400%	60%	75%	58%	Zinnanti *et al.,* 2009 [4]
Murine classic MSUD, P2			250%	40%	70%	70%	Zinnanti *et al.,* 2009 [4]
Murine classic MSUD (*Bckdha-/-*)*,* P4	802%	137%	929%	53%	113%	68%	Present study
**Average Across Studies**	**na**	**na**	**807%**	**45%**	**65%**	**54%**	
Range			*250-1400%*	*30-60%*	*19-113%*	*20-70%*	

*Not separable from threonine in this study.

Abbreviations: 2KG, 2-ketoglutaric acid; 2KIC, 2-ketoisocaproic acid; DOL, day of postnatal life; GABA, 4-aminobutyric acid; MSUD, maple syrup urine disease; na, not applicable; P, postnatal day.

Table S6. Within-Cohort and Cross-Cohort Correlations for Key Brain Metabolite Pairs

Metabolite Pair	Cohort	n	Pearson R^2	Pearson p	Spearman ρ*	Spearman p
**2KIC vs. Leucine**						
	P4 WT	3	0.437	0.5405	0.500	0.6667
	P4 MSUD	3	0.984	0.0812	1.000	0.0000
	P140 WT	3	0.960	0.1280	-0.866	0.3333
	P140 A-BiP-B	3	na	na	na	na
	**All cohorts (pooled)**	**12**	**0.998**	**0.0000**	**0.737**	**0.0062**
**2KG vs. Glutamate**						
	P4 WT	3	0.161	0.7372	-0.500	0.6667
	P4 MSUD	3	0.344	0.6007	0.500	0.6667
	P140 WT	3	0.935	0.1644	-1.000	0.0000
	P140 A-BiP-B	3	0.206	0.7000	-0.500	0.6667
	**All cohorts (pooled)**	**12**	**0.888**	**0.0000**	**-0.909**	**0.0000**
**2KG vs. Aspartate**						
	P4 WT	3	0.179	0.7217	0.500	0.6667
	P4 MSUD	3	0.774	0.3153	-0.500	0.6667
	P140 WT	3	0.429	0.5456	0.500	0.6667
	P140 A-BiP-B	3	0.139	0.7564	0.500	0.6667
	**All cohorts (pooled)**	**12**	**0.795**	**0.0001**	**-0.839**	**0.0006**

*Correlations were computed in brain tissue across four cohorts: P4 WT (n=3), P4 MSUD untreated (n=3), P140 WT (n=3), and P140 MSUD A-BiP-B-treated (n=3). Within-cohort correlations (n=3) are underpowered by design; they are presented for transparency to illustrate the extent to which cross-cohort correlations reflect between-group separation versus continuous within-group covariance. Cross-cohort (pooled) values correspond to those reported in Figure 3.

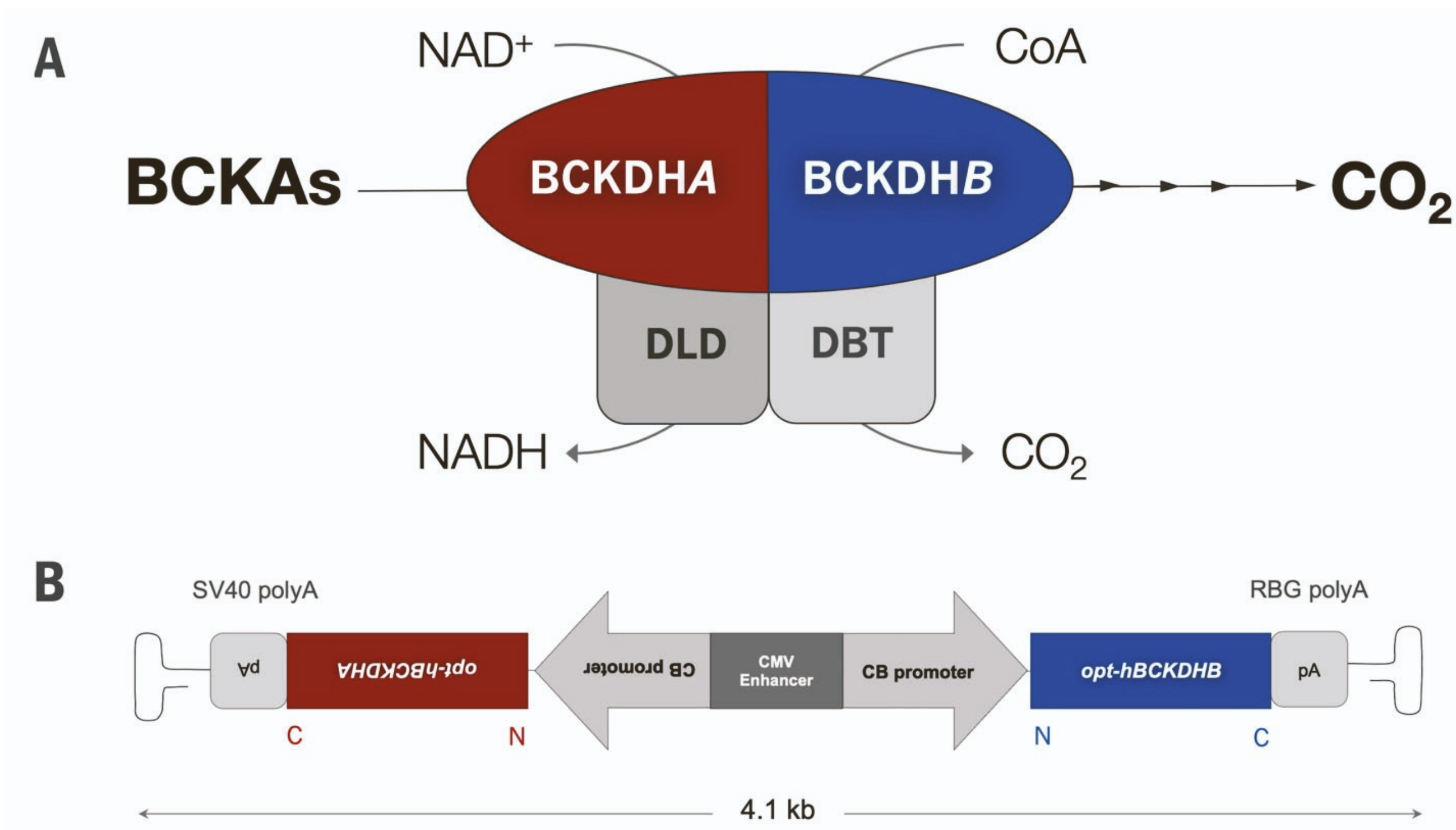

Figure S1. Rationale and design of *BCKDHA-BCKDHB* dual gene replacement vector. (**A**) Branched-chain α-ketoacids (BCKAs) are transamination (BCAT) derivatives of branched-chain amino acids (BCAAs) that undergo oxidative decarboxylation by branched-chain α-ketoacid dehydrogenase complex (BCKDH), which consists of BCKDHA, BCKDHB, DLD, and DBT subunits (CoA, coenzyme-A; NAD+/H, oxidized/reduced nicotinamide adenine dinucleotide). (**B**) Schematic of the dual-gene construct co-expressing codon-optimized (opti) human (h) *BCKDHA* and *BCKDHB* (h*A*-BiP-h*B*).

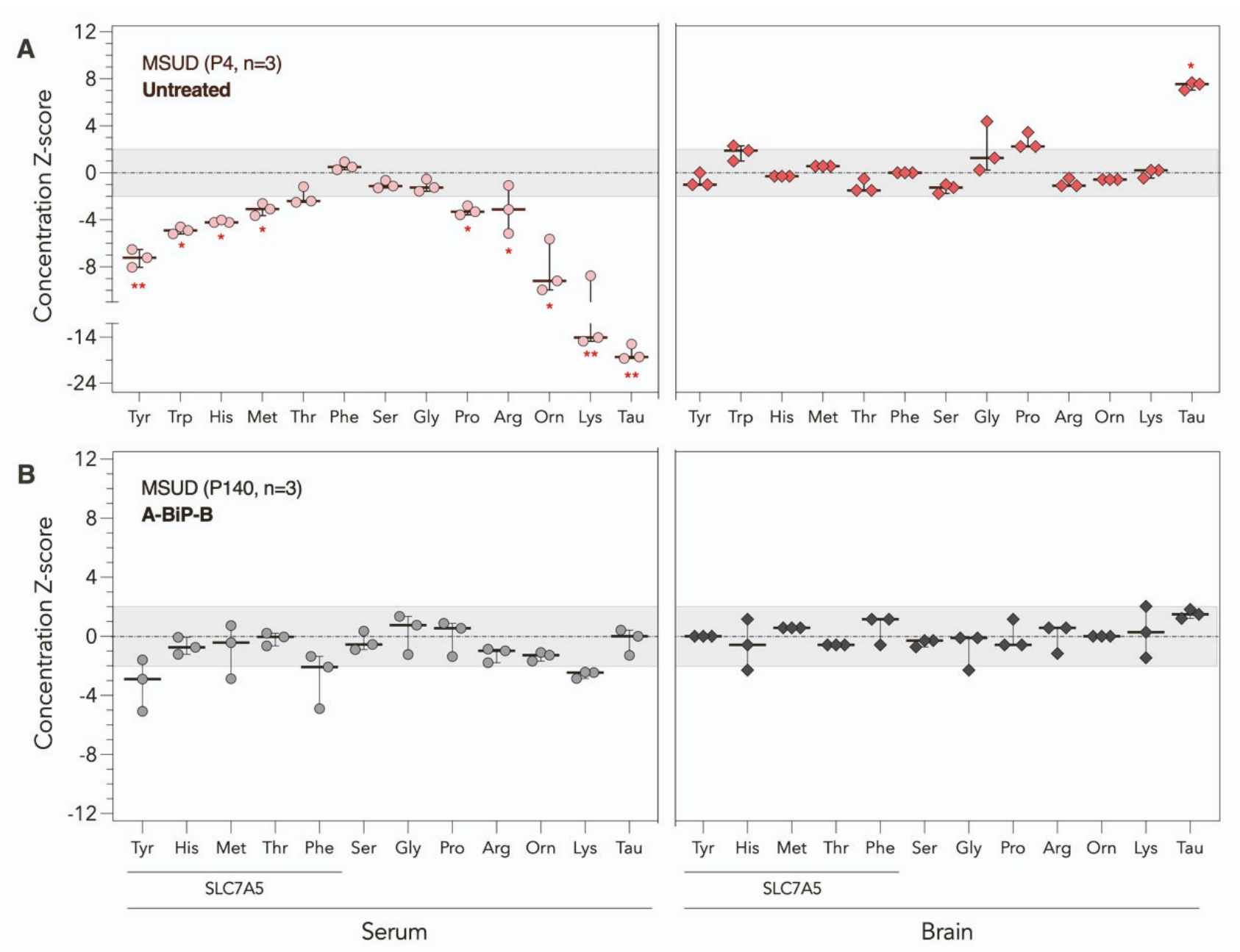

Figure S2. Amino acids in serum and brain of untreated and vector-treated *Bckdha-/-* mice. Left and right panels depict amino acid concentration z-scores in serum (circles) and brain (diamonds), respectively, of untreated P4 *Bckdha-/-* mice (panel A) and P140 *Bckdha-/-* mice treated with dual-gene therapy (A-BiP-B; panel B). **(A)** Most non-BCAA amino acids were depleted from serum of intoxicated *Bckdha-/-* mice (red), including substrates that normally compete with BCAAs for cerebral uptake via the selective large neutral amino acid transporter (SLC7A5). In contrast, most non-BCAA amino acids were unchanged in brain, with the notable exceptions of Glu and Gln, as well as Tau, which was increased 26% (q = 0.013) relative to wild-type brain. **(B)** All amino acid changes returned to the WT range in response to dual-gene therapy (gray). Multiple, unpaired, two-tailed t tests with Welch correction, FDR 5%: *q<0.05; **<0.01. *Abbreviations:* Arg, arginine; Gly, glycine; His, histidine; Lys, lysine; Met, methionine; Orn, ornithine; Phe, phenylalanine; Pro, proline; Ser, serine; Tau, taurine; Thr, threonine; Trp, tryptophan; Tyr, tyrosine.

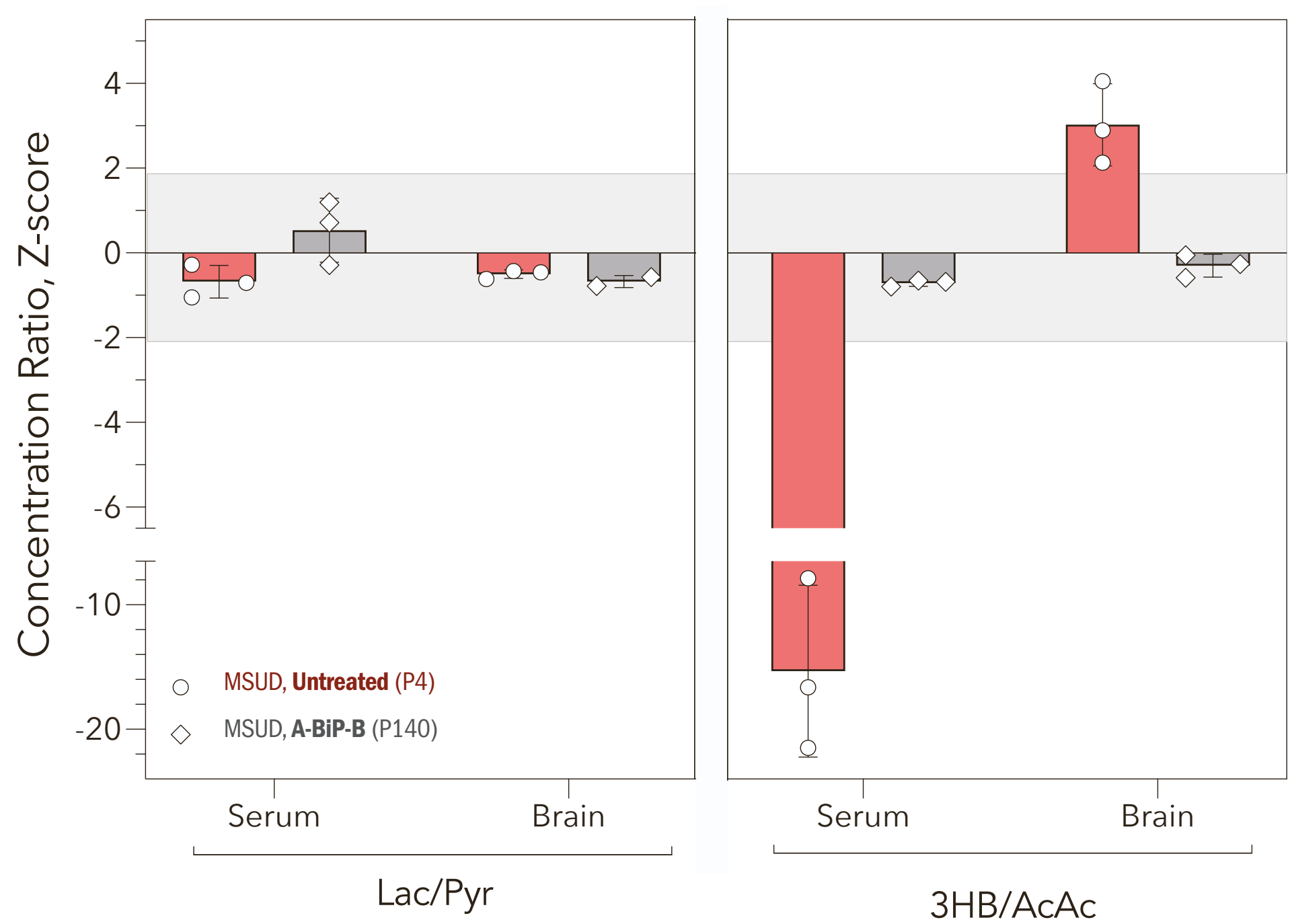

Figure S3. Cytosolic and mitochondrial redox pairs in serum and brain of untreated and vector-treated *Bckdha-/-* mice. The ratio of lactate to pyruvate (Lac/Pyr) and 3-hydroxybutyrate to acetoacetate (3HB/AcAc) is influenced by the ratio of oxidized (NAD^+) to reduced (NADH) nicotinamide adenine dinucleotide and therefore serves as a proxy for the redox state of cytosol and mitochondria, respectively. Cytosolic redox relationships (*left panel*) were undisturbed in serum and brain of P4 MSUD mice (red bars). In contrast, MSUD intoxication was associated with divergent mitochondrial redox markers in blood as compared to brain. These results signify an oxidized state of mitochondria in liver (the main site of ketone body synthesis) and reduced state of mitochondria in brain. These changes might relate to disturbances of the malate-aspartate shuttle, and returned to the WT range in response to dual-gene therapy (A-BiP-B; gray bars). Data are plotted as z-scores in relation to age-matched wild type mice.

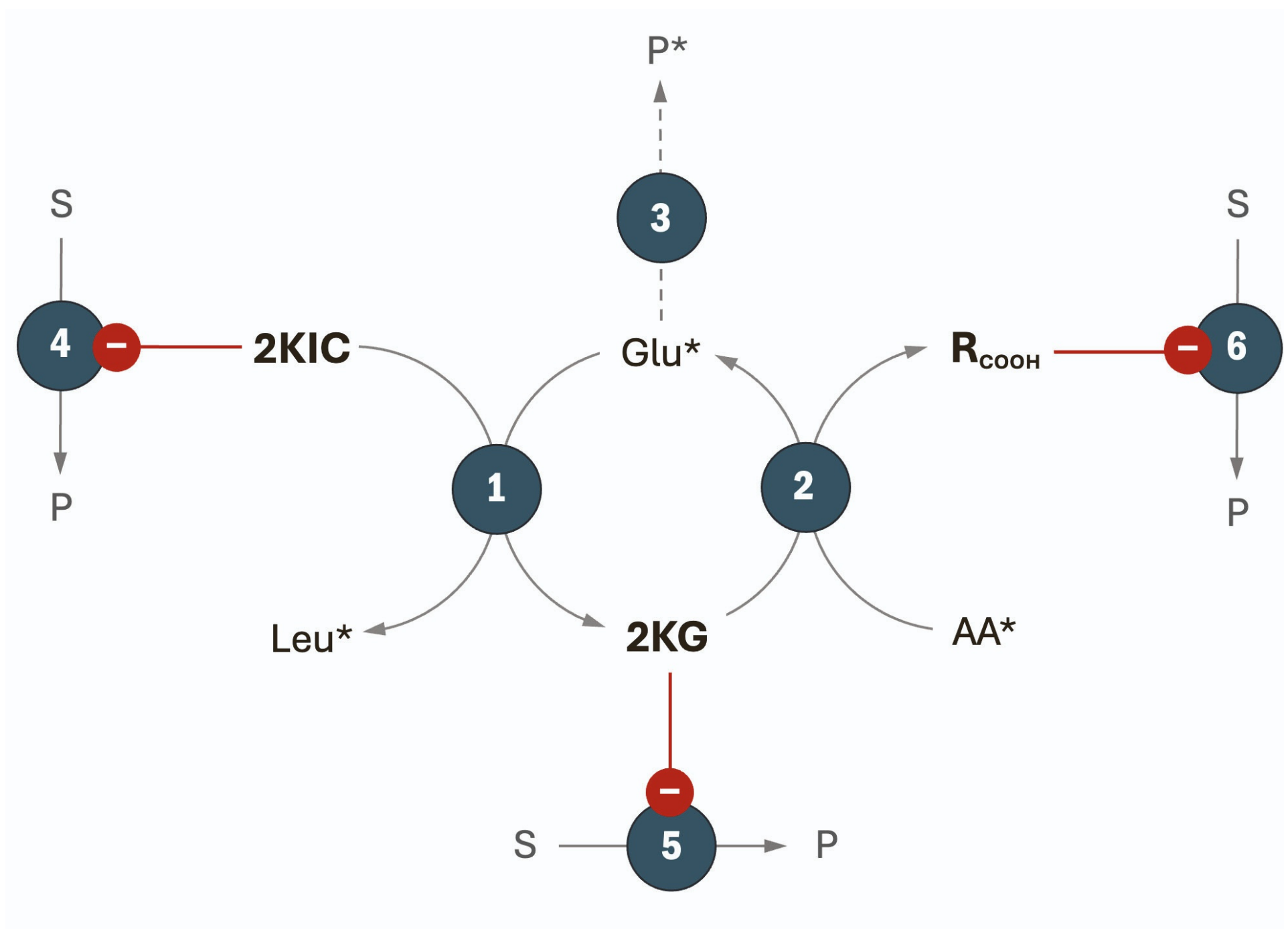

Figure S4. Mechanisms of 2-ketoisocaproate toxicity. 2KIC is the central driver of neurological intoxication in MSUD and acts by at least six potential mechanisms: (1) Reversal of net chemical flux through BCATs, depleting tissue Glu to form excess 2KG; (2) Alteration of the 2KG/Glu ratio biases flux through other transaminases; (3) Reduced tissue Glu can become rate-limiting for biosynthesis of important neural substrates such as Gln and GABA; and (4-6) Elevated tissue concentrations of 2KIC, 2KG, and various transaminase substrates (R_{COOH}) have the potential to directly inhibit other enzyme systems. Asterisks (*) designate a NH_2 group. *Abbreviations:* 2KG, 2-ketoglutarate; 2KIC, 2-ketoisocaproate; AA, amino acid; Glu, glutamate; Leu, leucine; P, product; R_{COOH}, carboxylic acid; S, substrate.

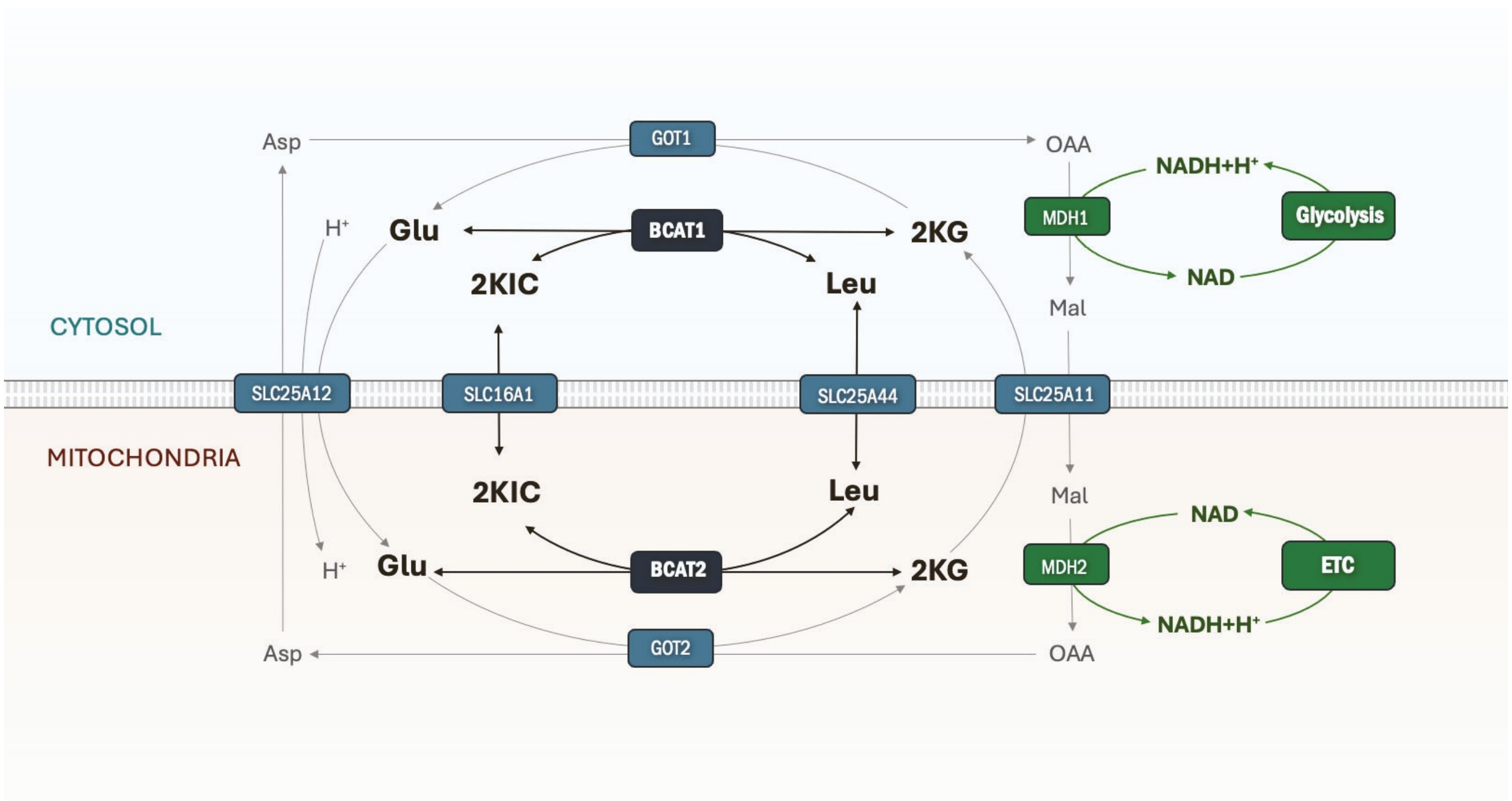

Figure S5. Branched-chain amino acid metabolism and the malate-aspartate shuttle. Coordinated nitrogen exchanges by BCAT1 and BCAT2 contribute to operation of the malate-aspartate shuttle, which normally transfers reducing equivalents formed by glycolysis into the mitochondrial matrix to support ATP production. *Abbreviations:* 2KG, 2-ketoglutarate; 2KIC, 2-ketoisocaproate; Asp, aspartate; ATP, adenosine triphosphate; BCAT, branched-chain aminotransferase; ETC, electron transport chain; Glu, glutamate; H^+, hydrogen ion; Leu, leucine; Mal, malate; NAD(H), oxidized (and reduced) nicotinamide adenine dinucleotide; OAA, oxaloacetate. Enzymes and transporters are abbreviated according to International Protein Nomenclature Guidelines.

Supplemental Methods

Vector construction

The AAV9-A-BiP-B genome (4.1 kb) was flanked by AAV2 ITRs and contained codon-optimized human **BCKDHA** and **BCKDHB** coding sequences. The bidirectional cassette was built on a central CMV immediate early enhancer with dual chicken β-actin promoters in opposing orientation. Transcripts terminated with an SV40 polyadenylation signal (hBCKDHA) and rabbit β-globin polyadenylation signal (hBCKDHB). The construct was packaged in recombinant AAV9 capsids and formulated in buffered aqueous suspension for IV delivery.

Nucleic acid assays

DNA and RNA were extracted from brain using the AllPrep DNA/RNA Mini kit (Qiagen, Cat. No. 80204). Viral genome copy number was determined by ddPCR (QX200, Bio-Rad) with the following primer/probe sets:

- **opti-hBCKDHA**: forward 5’-AGCAGGATCACCCTATCAGC-3’; reverse 5’-TCGAAGGCCTCCATCACTTT-3’; probe 5’-CCGGCGGCTCTGCTTCCTCCA-3’
- **Endogenous *Bckdha***: Thermo Fisher TaqMan Assay ID Mm00476112_m1
- **Reference (*Tbp*)**: Thermo Fisher TaqMan Assay ID Mm01277042_m1
- **Housekeeping gene (*Tfrc*)**: Thermo Fisher Cat. No. 4458367

RNA was reverse-transcribed with High Capacity cDNA Reverse Transcription Kit (Fisher, Cat. No. 43-688-13). Relative expression of opti-hBCKDHA was normalized to *Tbp* and endogenous *Bckdha*.

Tissue processing

Mice were transcardially perfused with ice-cold PBS. Brains were dissected, sectioned into small pieces, snap-frozen in liquid nitrogen, and stored at –80 °C until analysis.

Metabolite quantitation

~10 mg brain tissue was homogenized in water to yield a 10 mg/mL mixture. Stable-isotope internal standards were added to serum and brain homogenates.

- **Amino acids** were quantified by liquid chromatography–tandem mass spectrometry.
- **Organic acids** were quantified by capillary gas chromatography–mass spectrometry in selected ion monitoring mode, using positive electron impact ionization and stable isotope dilution.

Serum analytes are reported in µmol/L. Brain amino acids are reported in nmol/g tissue and organic acids in µmol/g tissue.

Targeted mass-action analysis

Metabolite data were evaluated according to the law of mass action. Equilibria included:

PSAT1: Glutamate + 3-Phosphopyruvate ⇌ 2KG + Phosphoserine → Serine (*Eqn S1*)

GLUL: Glutamate + ATP + NH_3 ⇌ Glutamine + ADP + Pi (*Eqn S2*)

GAD1: Glutamate ⇌ GABA + CO_2 (*Eqn S3*)

Linear or non-linear fits were chosen according to data shape. Goodness-of-fit was reported as R^2 and, when relevant, the standard deviation of the residuals (Sy.x).

Statistics

All analyses were performed in Prism 10 (GraphPad, San Diego). Group comparisons used unpaired, two-tailed Welch's t tests (assuming independent variances). To maximize stringency, p values were corrected for multiple comparisons across all analytes within each age x compartment comparison (27 metabolites per comparison) using the two-stage linear step-up method of Benjamini, Krieger, and Yekutieli (false discovery rate set at 5%). A q value ≤0.05 was considered a discovery. Effect sizes are reported as Hedges' g (bias-corrected standardized mean difference). Standard deviations are provided alongside SEM in all tables. Metabolite ratios were log-transformed prior to testing. Full statistical methods are described in the main text.

Graphical conventions:

- Data are presented as mean ± SEM.
- z-scores were used for selected figures and were calculated as: z = (value − WT mean) / WT SD. Gray shading indicates ±2 SD (approximately 95%) and ±3 SD (approximately 99.7%) relative to age-matched wild-type controls.
- Significance is annotated as *q≤0.05, **q≤0.01, ***q≤0.001

References

1. McKenna, M.C. (2007). The glutamate-glutamine cycle is not stoichiometric: fates of glutamate in brain. J Neurosci Res *85*, 3347–3358. 10.1002/jnr.21444.
2. Prensky, A.L., and Moser, H.W. (1966). Brain lipids, proteolipids, and free amino acids in maple syrup urine disease. J Neurochem *13*, 863–874.
3. Dodd, P.R., Williams, S.H., Gundlach, A.L., Harper, P.A., Healy, P.J., Dennis, J.A., and Johnston, G.A. (1992). Glutamate and gamma-aminobutyric acid neurotransmitter

systems in the acute phase of maple syrup urine disease and citrullinemia encephalopathies in newborn calves. J Neurochem *59*, 582–590.

4. Zinnanti, W.J., Lazovic, J., Griffin, K., Skvorak, K.J., Paul, H.S., Homanics, G.E., Bewley, M.C., Cheng, K.C., Lanoue, K.F., and Flanagan, J.M. (2009). Dual mechanism of brain injury and novel treatment strategy in maple syrup urine disease. Brain *132*, 903–918. awp024 [pii]
10.1093/brain/awp024.
